# Cyclin E overexpression in the *Drosophila* accessory gland induces tissue dysplasia

**DOI:** 10.3389/fcell.2022.992253

**Published:** 2023-01-10

**Authors:** Maria Molano-Fernández, Ian D. Hickson, Héctor Herranz

**Affiliations:** ^1^ Department of Cellular and Molecular Medicine, University of Copenhagen, Copenhagen, Denmark; ^2^ Department of Cellular and Molecular Medicine, Center for Chromosome Stability and Center for Healthy Aging, University of Copenhagen, Copenhagen, Denmark

**Keywords:** *Drosophila*, cyclin E, cancer, DNA damage, mitochondria, accessory gland, polyploidy

## Abstract

The regulation of the cell division cycle is governed by a complex network of factors that together ensure that growing or proliferating cells maintain a stable genome. Defects in this system can lead to genomic instability that can affect tissue homeostasis and thus compromise human health. Variations in ploidy and cell heterogeneity are observed frequently in human cancers. Here, we examine the consequences of upregulating the cell cycle regulator Cyclin E in the *Drosophila melanogaster* male accessory gland. The accessory gland is the functional analog of the human prostate. This organ is composed of a postmitotic epithelium that is emerging as a powerful *in vivo* system for modelling different aspects of tumor initiation and progression. We show that Cyclin E upregulation in this model is sufficient to drive tissue dysplasia. Cyclin E overexpression drives endoreplication and affects DNA integrity, which results in heterogeneous nuclear and cellular composition and variable degrees of DNA damage. We present evidence showing that, despite the presence of genotoxic stress, those cells are resistant to apoptosis and thus defective cells are not eliminated from the tissue. We also show that Cyclin E-expressing cells in the accessory gland display mitochondrial DNA aggregates that colocalize with Cyclin E protein. Together, the findings presented here show that Cyclin E upregulation in postmitotic cells of the accessory gland organ causes cellular defects such as genomic instability and mitochondrial defects, eventually leading to tissue dysplasia. This study highlights novel mechanisms by which Cyclin E might contribute to disease initiation and progression.

## Introduction

Cancer is a complex disease driven by an accumulation of mutations affecting different cellular functions known as the hallmarks of cancer. Collectively, these hallmarks cooperate to drive tumor progression and malignancy, and include processes such as uncontrolled proliferation, resisting apoptosis, genome instability and deregulated metabolism (Hanahan and Weinberg, 2011). Central alterations promoting disease progression are gain of function mutations in oncogenes, which frequently affect the function of cell cycle regulators. Consequently, cancer cells have a highly dysregulated cell cycle that leads to uncontrolled cell proliferation.

Cell proliferation is one of the most fundamental processes in living organisms. Before cells divide, they must replicate and segregate their genome faithfully to ensure the transmission of an intact copy of their genome to the resulting daughter cells. Thus, the cell cycle must be tightly regulated to prevent errors in these processes that could result in genomic instability. In addition to potential errors during cell cycle progression, cells are continually exposed to external insults that threaten the stability of their genome. To counteract internal and external threats, cells have evolved checkpoints that operate across the cell cycle and can detect and offset those threats. They sense defects in DNA integrity and react by slowing down or arresting the cell cycle to allow DNA repair, or by promoting cell death when DNA repair is unsuccessful.

Defects in cytokinesis, the last step of mitosis, can result in whole genome duplication and tetraploidy. Studies in experimental models have shown that whole genome duplication can precede the generation of aneuploid cells and tumorigenesis, and is a widespread feature of human cancers (Bielski et al., 2018; Lens and Medema, 2019). Tetraploid cells are genetically unstable and, when they divide, they can form multipolar spindles resulting in segregation errors and aneuploidy. To counteract that, cells possess systems to detect and prevent the proliferation of these cells (Ganem and Pellman, 2007; Ganem et al., 2014; Gerlach et al., 2018; Gerlach and Herranz, 2020). Although some of the mechanisms utilized by tumor cells to bypass these tumor suppressive mechanisms have been identified (Ganem and Pellman, 2007; Ganem et al., 2014; Gerlach et al., 2018; Gerlach and Herranz, 2020), we are still far from understanding the oncogenic signals driving tumorigenesis in cells with cytokinesis failure.

Cyclin E is a central cell cycle regulator commonly amplified in human cancer (Macheret and Halazonetis, 2015). Cyclin E levels oscillate during the cell cycle and are controlled by transcription and protein degradation. In normal mitotic cells, Cyclin E is expressed in G1. Cyclin E protein is active at the G1/S boundary, where it associates with CDK2 to stimulate G1/S progression. Cyclin E undergoes ubiquitin-mediated degradation throughout S-phase (Vermeulen et al., 2003; Siu et al., 2012). Cyclin E overexpression alters replication dynamics and promotes DNA replication stress, which is a source of genomic instability. In addition to its functions in mitotic cell cycles, studies in *Drosophila* showed that Cyclin E is crucial in endoreplicative cycles (Lilly and Spradling, 1996; Follette et al., 1998; Lilly and Duronio, 2005). Endocycles alternate S and gap phases and, in contrast to mitotic cycles, do not involve any mitosis. Endoreplicative cycles lead to the formation of polyploid cells that play central roles in development and homeostasis, and can contribute to cancer (Fox and Duronio, 2013).

In this study, we have investigated the effects of Cyclin E upregulation in the *Drosophila* male accessory gland. This organ is the functional analog of the human prostate. It plays a central role in fertility and produces most of the seminal fluid components. The *Drosophila* accessory gland contains two types of secretory cells: namely, main cells, the most abundant cell type, and secondary cells, present in the distal tip of this organ (Supplementary Figure S1). Previous analyses have shown that tumorigenesis in both organs, namely the human prostate and the *Drosophila* accessory gland, can be driven by similar mechanisms (Ito et al., 2014; Wilson et al., 2017; Rambur et al., 2020; Rambur et al., 2021). The accessory gland is emerging as a useful model to study different aspects related to cancer initiation and progression (Ito et al., 2014; Wilson et al., 2017; Rambur et al., 2020; Rambur et al., 2021; Box et al., 2022). The adult accessory gland is a postmitotic organ. It is composed of binucleated epithelial cells that do not proliferate during the life of the adult male (Box et al., 2022). *Drosophila* males contain two accessory gland lobes, each consisting of a layer of epithelial cells that surround the lumen of the gland, and an enclosing muscle layer (Ravi Ram and Wolfner, 2007; Wilson et al., 2017). The development of this organ takes place during larval and pupal stages. Throughout the developmental period, this organ grows by standard mitotic divisions of diploid cells until 50 h after puparium formation. At this point, cells undergo cell cycle arrest and hence stop proliferating. Between 55 and 60 h after puparium formation, cells go through a final round of mitosis without cytokinesis, which leads to the formation of binucleated tetraploid cells. This is followed by an additional round of DNA replication leading to the formation of binucleated cells in which each nucleus has a 4C DNA content (Taniguchi et al., 2014; Box et al., 2022).

In this study, we show that Cyclin E overexpression in the *Drosophila* male accessory gland leads to tissue dysplasia. These dysplastic organs are overgrown and exhibit cell heterogeneity with cells of variable shapes and sizes. We also show that persistent expression of Cyclin E triggers endoreplication and polyploidy. This is accompanied by high levels of DNA damage and hence genomic instability. Interestingly, previous studies in *Drosophila* showed that continuous Cyclin E expression represses endoreplication cycles in salivary glands and in ovary nurse cells (Lilly and Spradling, 1996; Follette et al., 1998). The analysis presented here reveals that Cyclin E upregulation can lead to different cellular outcomes in a context-dependent manner. Finally, we show that cells expressing Cyclin E display extranuclear DNA aggregates that colocalize with mitochondrial markers and suggest that this cell cycle regulator could control mitochondrial DNA copy number and hence mitochondrial function.

## Results

### Cyclin E upregulation in the accessory gland increases organ size

**FIGURE 1:**
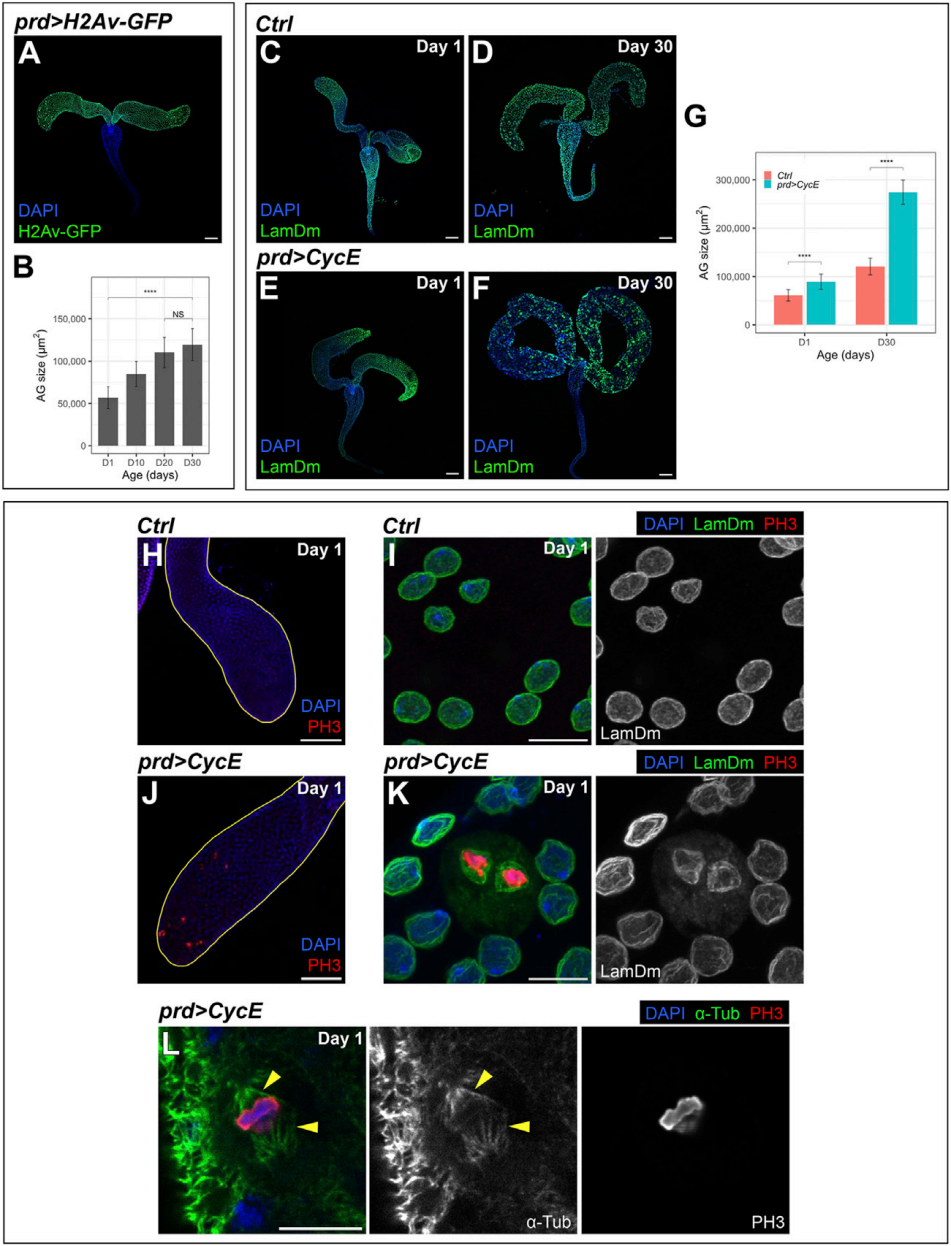
Cyclin E upregulation drives a size increase in the accessory gland. **(A)** Confocal image of an accessory gland that expresses H2Av-GFP under *prd-Gal4* control. DAPI and H2Av-GFP are shown in blue and green, respectively. Scale bar, 100 µm. **(B)** Quantification of *yw* accessory gland (AG) size at days 1, 10, 20 and 30. Data shown are mean ± SD. Statistical analysis was performed using a one-way ANOVA followed by a Tukey’s HSD test. Only the relevant significant changes are shown. ****, *p* < .0001. NS, non-significant. **(C–G)** Confocal images of 1-day-old and 30-day-old control (*prd-Gal4/+*) [**(C,D)**, respectively] and *prd > CycE* [**(E,F)**, respectively] accessory glands, and their size quantification **(G)**. Note that images in **(C,E)** were re-sized to have the same magnification as **(D,F)** *.* In **(C–F)**, DAPI and LamDm are shown in blue and green, respectively. Data shown in **(G)** are mean ± SD. Statistical analysis was performed using a two-way ANOVA followed by a Tukey’s HSD test. Only the relevant significant changes are shown. ****, *p* < .0001. Scale bars, 100 µm. **(H,J)** Confocal images of 1-day-old control (*prd-Gal4/+*) **(H)** and *prd > CycE* **(J)** glands stained with anti-PH3. Accessory glands are outlined in yellow. DAPI and PH3 are shown in blue and red, respectively. Scale bars, 100 µm. **(I,K)** Confocal images of 1-day old control (*prd-Gal4/+*) **(I)** and *prd > CycE* **(K)** glands labeled with anti-Lamin Dm and anti-PH3. DAPI, LamDm and PH3 are shown in blue, green, and red, respectively. Scale bars, 10 µm. **(L)** Confocal image of a 1-day-old *prd > CycE* gland stained with anti-alpha-Tubulin (α-Tub) and anti-PH3. The yellow arrowheads point to the mitotic spindle. DAPI, α-Tub and PH3 and shown in blue, green, and red, respectively. Scale bar, 10 μm.

The male accessory gland of *Drosophila* (Figure 1A, Supplementary Figure S1) is the functional equivalent of the mammalian prostate. The adult accessory gland has a characteristic structure that is formed by a layer of binucleated epithelial cells. Similar to the human prostate, this organ expands during adulthood (Figure 1B). Cyclin E is an oncogene, and Cyclin E upregulation induces premature entry into S-phase and subsequent replicative stress, which is a central feature of human tumors (Macheret and Halazonetis, 2015). Consistently, Cyclin E upregulation correlates with uncontrolled growth and cell proliferation (Hwang and Clurman, 2005). We used the binary Gal4-UAS system (Brand and Perrimon, 1993) to determine the effects of Cyclin E upregulation on the accessory gland. The paired-Gal4 (prd-Gal4) line is expressed throughout this organ (Figure 1A) and can be utilized to drive Cyclin E expression. We observed that Cyclin E expression led to an increase in tissue size, which was greater in magnitude than the one observed in normal glands (Figures 1C–G). During this analysis, we overexpressed Cyclin E throughout development and adulthood. To determine the partial contribution of each of those stages to the increase in organ size, we analyzed glands in males recently eclosed (day 0). We did not find a size difference between glands expressing Cyclin E and control glands at day 0 (Supplementary Figure S2). This suggests that Cyclin E-driven tissue overgrowth occurs during adulthood. We performed the reciprocal experiment and analyzed glands depleting Cyclin E. Contrasting with the increase in gland size observed in glands upregulating Cyclin E (Figures 1C–G), we did not detect overt defects in glands expressing Cyclin E-RNAi (Supplementary Figure S3). This suggests that Cyclin E does not play a major role controlling normal size or tissue organization in male accessory glands.

We studied whether the increase in organ size observed in Cyclin E-expressing organs was caused by cell proliferation. To test this, we labeled control and Cyclin E-expressing glands with the mitotic marker phospho-histone 3 (PH3). We also labeled those organs with the nuclear envelope marker, Lamin Dm. The nuclear envelope is a dynamic structure, and changes in the nuclear envelope can be used to detect the presence of mitotic cells. The nuclear envelope disassembles to allow chromosome segregation. After mitosis, it reassembles to compartmentalize the nuclear DNA and separate it from the cytoplasm (Dey and Baum, 2021). We did not detect mitotic cells in control or Cyclin E-expressing glands analyzed at different ages (Figures 1H, I; Supplementary Figure S4), which suggests that, as observed in normal glands (Box et al., 2022), the increase in size in Cyclin E-expressing glands is not caused by cell proliferation. Interestingly, we detected some scattered PH3-positive cells in Cyclin E-expressing glands dissected from 1-day old males (Figures 1J, K). Antibodies detecting alpha-Tubulin revealed the presence of the mitotic spindle in those cells (Figure 1L). Furthermore, the nuclear envelope in those cells presented a diffuse appearance, which is characteristic of mitotic cells. The nuclear envelope in PH3-positive cells contrasted with the defined signal surrounding the nuclear DNA present in PH3-negative cells (Figures 1I, K). Day 1 control glands were negative for PH3 (Figures 1H, I). Mitotic cells were only observed in the most distal part of the accessory gland, which corresponds to the region where secondary cells reside (Figure 1J, Supplementary Figure S1). Secondary cells can be differentiated from main cells because their apical surface is smaller than the one in surrounding main cells, as revealed by the subapical marker DE-Cadherin (Supplementary Figure S1). Mitotic cells in Cyclin E-expressing glands exhibited the characteristic features of secondary cells (Supplementary Figure S1), which, together with the topological localization of mitosis (distal region of the gland), suggest that they correspond to secondary cells.

Cyclin E-expressing glands increased size from day 1 to day 30 (Figures 1E–G). The absence of mitosis after day 1 supports the notion that the increase in organ growth induced by Cyclin E is independent of cell proliferation. The mitoses observed in 1-day old males appear to be specific of secondary cells and could correspond to stalled mitoses from the last mitotic cycle during the development of the organ. This is consistent with previous studies showing that Cyclin E upregulation in human cells can impair mitotic progression, thus causing mitotic delays (Keck et al., 2007; Bagheri-Yarmand et al., 2010). Taken together, these observations suggest that Cyclin E upregulation is not able to induce a sustained level of mitotic cell cycles in the binucleated cells of the accessory gland that could explain the increase in gland size.

### Cyclin E induces endoreplication in the accessory gland

The results showing that the accessory gland grew robustly in the absence of cell proliferation (Figure 1; Supplementary Figure S4), led us to consider alternative mechanisms by which Cyclin E induced tissue overgrowth. The endocycle, or endoreplicative cycle, is characterized by continuous rounds of DNA replication without mitosis. This is a specialized kind of cell cycle in which cells alternate between consecutive G- and S-phases. Uncoupling DNA replication from mitosis allows for the expansion of the genomic content and the generation of polyploid cells. While polyploidy plays central roles in development and homeostasis, it can also have negative consequences on human health. Polyploidy is frequently observed in human cancers and correlates with malignancy and poor prognosis (Fox and Duronio, 2013; Coward and Harding, 2014; Bou-Nader et al., 2020).

**FIGURE 2:**
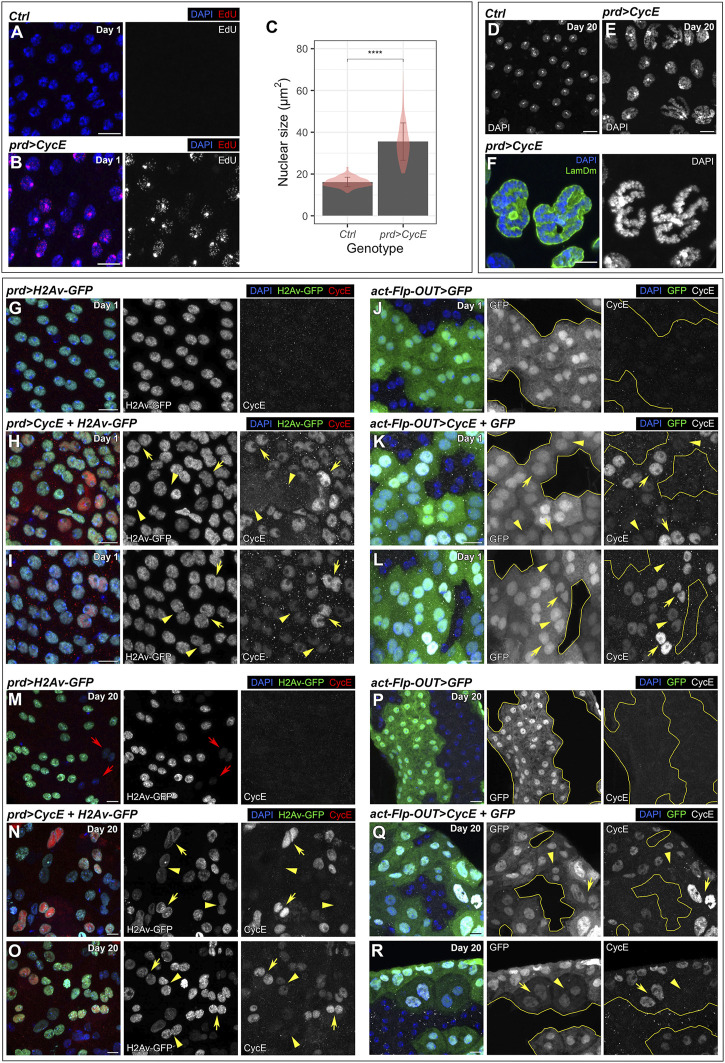
Cyclin E overexpression induces endoreplication. **(A,B)** Confocal images of control (*prd-Gal4/+*) **(A)** and *prd > CycE* **(B)** accessory glands at day 1 labeled with EdU. DAPI and EdU are shown in blue and red, respectively. Scale bars, 10 µm. **(C)** Quantification of nuclear size in control (*prd-Gal4/+*) and *prd > CycE* accessory glands at day 1. Data shown are mean ± SD. The bars are overlayed with violin plots to showcase the distribution of the data. Statistical analysis was performed using a Mann-Whitney test for unpaired data. ****, *p* < .0001. **(D–E)** Confocal images of cells in 20-day-old control (*prd-Gal4/+*) **(D)** and *prd > CycE* **(E)** accessory glands. DAPI is shown in grays. Scale bars, 10 µm. **(F)** Confocal image of nuclei with polytene chromosomes in *prd > CycE* accessory glands at day 20. DAPI and LamDm are shown in blue and green, respectively. Scale bar, 10 µm. **(G–I)** Confocal images of control (*prd > H2Av-GFP*) **(G)** and *prd > CycE + H2Av-GFP* **(H,I)** glands at day 1 stained with anti-CycE. In **(H,I)**, yellow arrowheads point to cells with absent or low Cyclin E levels in the nucleus, whereas yellow arrows point to cells with high levels of Cyclin E in the nucleus. DAPI, H2Av-GFP and CycE are shown in blue, green, and red, respectively. Note that the Gal80ts system was used in this experiment. Scale bars, 10 µm. **(J–L)** Confocal images of control (*act-Flp-OUT>GFP*) **(J)** and *act-Flp-OUT>CycE + GFP* **(K,L)** at day 1 stained with anti-CycE. Gal4-expressing cells can be identified by GFP, and the borders between GFP-positive and GFP-negative cells are outlined in yellow. In **(K,L)**, yellow arrowheads point to cells with absent or low Cyclin E levels in the nucleus, whereas yellow arrows point to cells with high levels of Cyclin E in the nucleus. DAPI, GFP and CycE are shown in blue, green, and grays, respectively. Scale bars, 10 µm. **(M–O)** Confocal images of control (*prd > H2Av-GFP*) **(M)** and *prd > CycE + H2Av-GFP* **(N,O)** glands at day 20 stained with anti-CycE. In **(M)**, red arrows point to cells with very low H2Av-GFP expression, possibly due to a lower expression of the Gal4 driver. In **(N,O)**, yellow arrowheads point to cells with absent or low Cyclin E levels in the nucleus, whereas yellow arrows point to cells with high levels of Cyclin E in the nucleus. DAPI, H2Av-GFP and CycE are shown in blue, green, and red, respectively. Note that the Gal80ts system was used in this experiment. Scale bars, 10 µm. **(P–R)** Confocal images of control (*act-Flp-OUT>GFP*) **(P)** and *act-Flp-OUT>CycE + GFP* **(Q,R)** at day 20 stained with anti-CycE. Gal4-expressing cells can be identified by GFP, and the borders between GFP-positive and GFP-negative cells are outlined in yellow. In **(Q,R)**, yellow arrowheads point to cells with absent or low Cyclin E levels in the nucleus, whereas yellow arrows point to cells with high levels of Cyclin E in the nucleus. DAPI, GFP and CycE are shown in blue, green, and grays, respectively. Scale bars, 10 µm.

To study if Cyclin E induced endoreplication, we incubated accessory glands dissected from 1-day-old and 20-day-old males with 5-Ethynyl-2′deoxyuridine (EdU). The EdU incorporation assay is an efficient method to label cells in S-phase. We did not detect EdU signal in control glands, suggesting that, at these ages, cells do not duplicate their DNA (Figure 2A; Supplementary Figure S5). In contrast, Cyclin E-expressing glands incorporated EdU (Figure 2B; Supplementary Figure S5), indicating that Cyclin E induces DNA replication. In most of the cells, the EdU signal was more intense in a nuclear region strongly labeled by DAPI that might correspond to the nucleolus. In accordance with the results obtained by EdU labeling, 1-day-old Cyclin E-expressing nuclei were larger than controls, suggesting that they have a higher DNA content (Figure 2C). Glands dissected at later time points revealed the presence of high nuclear heterogeneity, suggesting that different cells could have a variable DNA content (Figures 2D, E). A detailed analysis revealed the presence of cells in which the DNA organization resembled polytene chromosomes (Figure 2F). Polytene chromosomes are a special type of chromosomes formed by endoreplication. Newly formed chromatids in polytene chromosomes remain associated together forming a characteristic structure of 1 chromocenter and 5 arms, each of them corresponding to a chromosome arm. We identified nuclei displaying that organization in some Cyclin E-expressing cells in older glands (Figure 2F). Taken together, these data suggest that Cyclin E induces endoreplication in the binucleated cells of the accessory gland. This is consistent with previous observations (Leiblich et al., 2019). Contrasting with these results, previous reports have shown that continuous overexpression of Cyclin E in *Drosophila* salivary glands or ovarian follicle cells represses endocycling and hence limits the formation of polyploid cells (Calvi et al., 1998; Follette et al., 1998; Weiss et al., 1998; Shcherbata et al., 2004). These studies demonstrated that persistent Cyclin E accumulation has negative effects in endocycle progression and that periods with low Cyclin E levels are required for assembly of pre-replication complexes and subsequent cycles of DNA replication. To assess whether the levels of Cyclin E protein were oscillating when continuously expressed in the cells of the accessory gland, we labeled Cyclin E-expressing glands with antibodies detecting Cyclin E. We observed that Cyclin E levels varied between cells. We found that while some cells exhibited an intense Cyclin E signal in the nucleus, other cells presented lower levels and, in some cells, Cyclin E was not detected (Figures 2G–I, M–O). During that analysis, we coexpressed Cyclin E with a transgene driving the expression of nuclear GFP (*UAS-H2Av-GFP*) that can be used as a surrogate of Gal4 activity. We observed that Cyclin E levels did not correspond to the levels of Gal4 activity as visualized by the nuclear GFP transgene. Some cells positive for the nuclear GFP marker did not have detectable Cyclin E. Similarly, cells with high Cyclin E levels did not present similar nuclear GFP signal (Figures 2G–I, M–O). To further validate this observation, we monitored Cyclin E protein levels in Cyclin E-expressing GFP-labeled clones controlled by the actin-Gal4 driver. We obtained comparable results showing that Cyclin E levels varied independently of differences in Gal4 activity (Figures 2J–L, P–R). These results showed that, although Cyclin E was constantly expressed from a transgene, Cyclin E protein was under the control of post-transcriptional regulators that ensure an oscillatory pattern. This has been shown to be essential to support endoreplication and polyploidy (Calvi et al., 1998; Follette et al., 1998; Weiss et al., 1998).

### Cyclin E-driven DNA damage and genomic instability in the accessory gland

**FIGURE 3:**
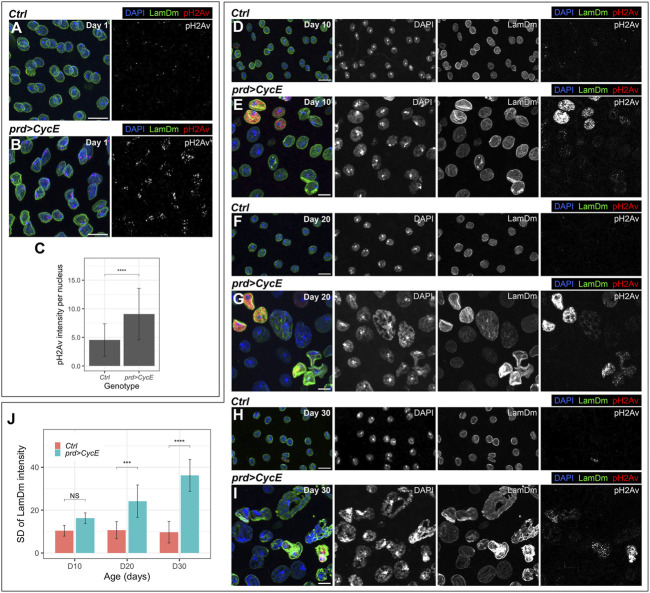
Cyclin E-driven DNA damage and genomic instability. **(A–C)** Confocal images of 1-day-old control (*prd-Gal4/+*) **(A)** and *prd > CycE* **(B)** accessory glands stained with anti-Lamin Dm and anti-pH2Av, and the corresponding pH2Av intensity quantification **(C)**. In **(A,B)**, DAPI, LamDm and pH2Av are shown in blue, green, and red, respectively. Data shown in **(C)** are mean ± SD. Statistical analysis was performed using a Mann-Whitney test for unpaired data. ****, *p* < .0001. Scale bars, 10 µm. **(D–I)** Confocal images of 10-day-old, 20-day-old and 30-day-old control (*prd-Gal4/+*) [**(D,F,H)**, respectively] and *prd > CycE* [**(E,G,I)**, respectively] accessory glands labeled with anti-Lamin Dm and pH2Av. DAPI, LamDm and pH2Av are shown in blue, green, and red, respectively. Scale bars, 10 µm. **(J)** Quantification of the standard deviation of Lamin Dm intensity of control (*prd-Gal4/+*) and *prd > CycE* glands at days 10, 20, and 30. Data shown are mean ± SD. Statistical analysis was performed using a two-way ANOVA followed by a Tukey’s HSD test. Only the relevant significant changes are shown. NS, non-significant. ***, *p* < .001, ****, *p* < .0001.

Typically, endocycling cells end S-phase and transition to G-phase before the entire genome is duplicated. Hence, S-phase truncation leads to the underrepresentation of late replicating sequences in polyploid cells (Gall et al., 1971; Hammond and Laird, 1985a; Hammond and Laird, 1985b; Lilly and Spradling, 1996). As a consequence of S-phase truncation, endocycling cells recurrently replicate the DNA in the presence of a high number or collapsed forks, resulting in persistent DNA damage (Leach et al., 2000). Interestingly, the extent of S-phase truncation is influenced by Cyclin E oscillatory dynamics (Lilly and Spradling, 1996; Doronkin et al., 2003). These observations led us to analyze whether Cyclin E overexpression could cause DNA damage and thus threaten genome stability in accessory glands. In *Drosophila*, the presence of double-strand breaks triggers the phosphorylation of the histone H2Av, and antibodies recognizing phospho-H2Av (pH2Av) can be used to identify the presence of DNA damage. Some pH2Av-positive foci were observed in control glands (Figure 3A). However, we observed an increase in pH2Av signal in Cyclin E-expressing cells, as compared to control glands (Figures 3B, C). This experiment revealed the presence of high levels of DNA damage in glands in which Cyclin E was upregulated, which could result from the accumulation of collapsed forks in endocycling cells.

DNA damage is a source of genetic instability typically present in tumors (Tubbs and Nussenzweig, 2017). The accumulation of DNA damage in Cyclin E-expressing cells might influence the endoreplication rate in subsequent rounds of DNA synthesis and thus the resulting DNA content. To analyze this hypothesis, we scrutinized how nuclei with persistent Cyclin E expression evolved with time. We analyzed control and cyclin E-overexpressing glands in 10, 20 and 30-day-old males. We observed that nuclei in 10-day-old cyclin E-overexpressing glands were bigger than their control counterparts (Figures 3D, E). This was exacerbated in 20-day-old and 30-day-old glands (Figures 3F–I). Importantly, nuclear sizes at these times points were highly heterogeneous. Moreover, the levels of DNA damage were also variable between different cells in those glands (Figures 3D–I). These results suggest that the accumulation of DNA damage induced by Cyclin E overexpression differentially affected the degree of DNA replication in different cells. This led to the presence of cells with variable DNA content that could eventually lead to genetic imbalances.

Additionally, even though nuclei in 10-day-old cyclin E-expressing glands generally maintain a round shape, they did acquire abnormal shapes at later stages (Figures 3D–I). Spherical nuclear shapes are determined to a great extent by the nuclear lamina (Webster et al., 2009). Strikingly, nuclei in 10-day-old cyclin E-expressing and especially older glands display heterogeneous Lamin Dm levels (Figures 3D–J). Alterations in Lamin Dm levels in cyclin E-expressing glands could contribute to the aberrations observed in nuclear shape. Notably, irregularities in nuclear size and shape are common in malignant tumors (Gisselsson et al., 2001; Zink et al., 2004).

### Aberrant epithelial organization in accessory glands upregulating Cyclin E

Malignant carcinomas are characterized by a pronounced disorganization in their epithelial architecture (Bilder, 2004; Wodarz and Nathke, 2007). We have shown that accessory gland cells expressing Cyclin E show a gradual increase in nuclear size and heterogeneity throughout their lifespan. These defects could have a direct impact in the organization and integrity of this epithelium.

**FIGURE 4:**
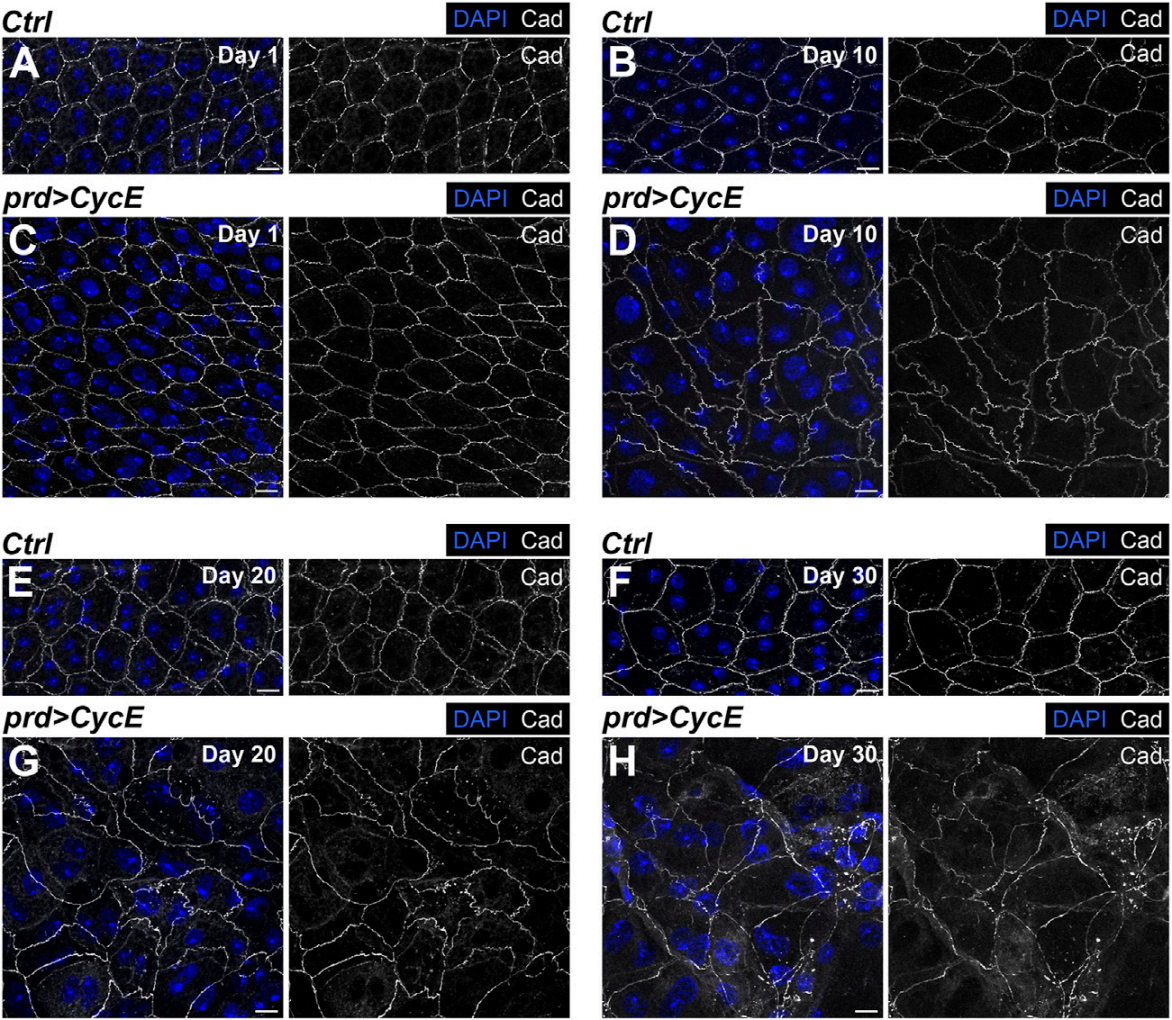
Cyclin E overexpression perturbs epithelial integrity. **(A–H)** Confocal images of 1-, 10-, 20- and 30-day-old control (*prd-Gal4/+*) [**(A,B,E,F)**, respectively] and *prd > CycE* glands [**(C,D,G,H)**, respectively] stained with anti-DE-Cadherin. DAPI and Cad are shown in blue and grays, respectively. Scale bars, 10 µm.

To determine potential changes in the epithelial organization, we labeled control and Cyclin E-expressing glands with the polarity marker DE-Cadherin. This protein localizes in the apical part of the epithelial cells of the accessory gland. It allowed us to visualize cellular shape and assess tissue organization. Control glands presented a regular hexagonal DE-Cadherin pattern at the different ages analyzed (Figures 4A, B, E, F). This pattern was perturbed in glands expressing Cyclin E (Figures 4C, D, G, H). Although 1-day-old Cyclin E-expressing glands showed a similar organization as the one observed in control organs (Figures 4A, C), 10-day-old cyclin E-expressing glands exhibited an abnormal pattern of DE-Cadherin deviating from the one observed in control glands, thus revealing defects in the organization of the epithelium (Figures 4B, D). As observed with nuclear sizes and shapes, these defects were intensified during aging and reveal a gradual accumulation of epithelial disorganization (Figures 4E–H).

### Cyclin E-expressing glands are resistant to apoptosis

We have shown that Cyclin E expression causes DNA damage (Figure 3). In *Drosophila*, induction of DNA damage by irradiation in the proliferating imaginal disc cells leads to the activation of the DNA damage response. This induces cell cycle arrest and the activation of an apoptotic cascade (Haynie and Bryant, 1977; Brodsky et al., 2000; Brodsky et al., 2004; Jaklevic and Su, 2004). The observation that Cyclin E-expressing cells had DNA damage, yet continued endoreplicating and growing, suggested that these cells suppressed their apoptotic program. This would be in agreement with previous studies showing that polyploid cells present a higher threshold for apoptotic induction and, in some cases, can block apoptosis in response to genotoxic stress (Mehrotra et al., 2008; Zheng et al., 2012; de Renty et al., 2014; Hassel et al., 2014).

We used markers of apoptosis to evaluate whether Cyclin E-expressing glands can activate the apoptotic cascade. Key elements of apoptosis are caspases. The most relevant caspases in *Drosophila* are death regulator Nedd2-like caspase (Dronc), which is a caspase 9-like initiator caspase, and death caspase 1 (Dcp-1) and death-related ICE-like caspase (DrICE), which are both caspase 3-like executioner caspases (Xu et al., 2009). Antibodies raised against cleaved human Caspase-3 detect Dcp-1 and DrICE proteins (Fan and Bergmann, 2010), and have proven useful in identifying apoptotic cells in *Drosophila*.

**FIGURE 5:**
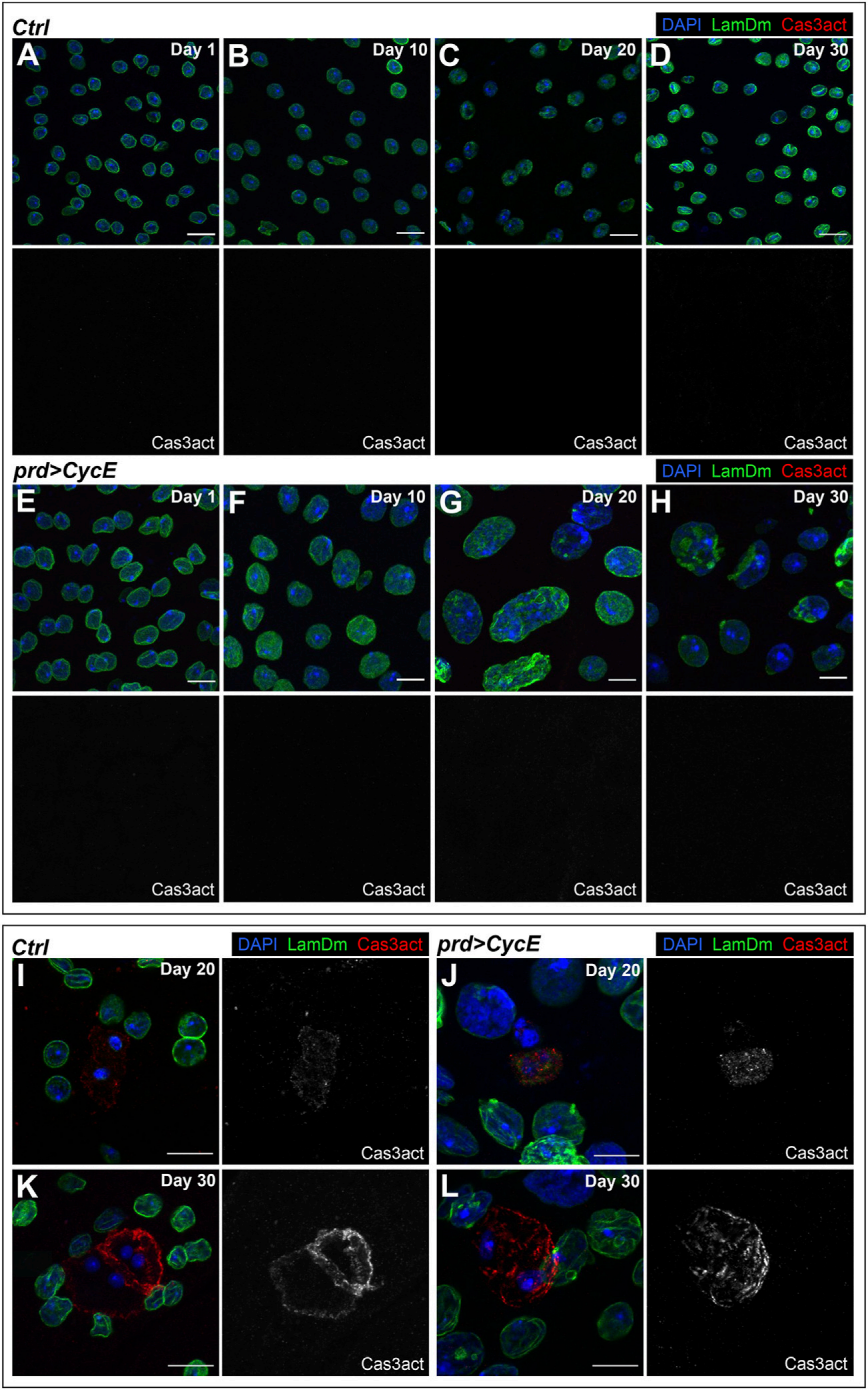
Polyploid cells with DNA damage bypass apoptosis. **(A–H)** Confocal images of 1-, 10-, 20- and 30-day-old control (*prd-Gal4/+*) [**(A–D)**, respectively] and *prd > CycE* [**(E–H)**, respectively] glands stained with anti-Lamin Dm and anti-cleaved Caspase 3 (Cas3act). DAPI, LamDm and Cas3act are shown in blue, green, and red, respectively. Scale bars, 10 µm. **(I–L)** Confocal images of apoptotic cells in 20-day-old and 30-day-old control (*prd-Gal4/+*) [**(I,K)**, respectively] and *prd > CycE* [**(J,L)**, respectively] accessory glands stained with anti-Lamin Dm and anti-cleaved Caspase 3. DAPI, LamDm and Cas3act are shown in blue, green, and red, respectively. Scale bars, 10 µm.

We dissected Cyclin E-expressing glands at different ages (day 1, 10, 20, and 30) and labeled those organs with anti-cleaved Caspase-3 antibodies. We did not detect apoptotic cells in 1-day-old or 10-day-old Cyclin E-expressing glands (Figures 5A, B, E, F). This, together with the observation that at those stages Cyclin E-expressing glands presented high DNA damage levels and endoreplication, suggests that endocycling cells expressing Cyclin E do not activate apoptosis in response to DNA damage. Discs overexpressing Cyclin E were labeled in parallel and used as positive controls (Supplementary Figure S6). Cyclin E overexpression in the imaginal discs is known to induce apoptosis (Shu and Deng, 2017). This allowed us to confirm that the antibody used worked efficiently and was able to detect apoptotic cells. Most of the glands dissected at days 20 and 30 yielded the same result and no apoptotic cells were observed (Figures 5C, D, G, H). However, we detected a few sporadic Cyclin E-expressing glands dissected at days 20 or 30 containing 2-3 cells positive for cleaved Caspase-3 (Figures 5J, L). Notably, analyses of control glands showed comparable results (Figures 5I, K), indicating that late apoptotic events in this organ could occur independently of Cyclin E expression.

### Cyclin E drives the accumulation of mitochondrial DNA in the accessory gland

**FIGURE 6:**
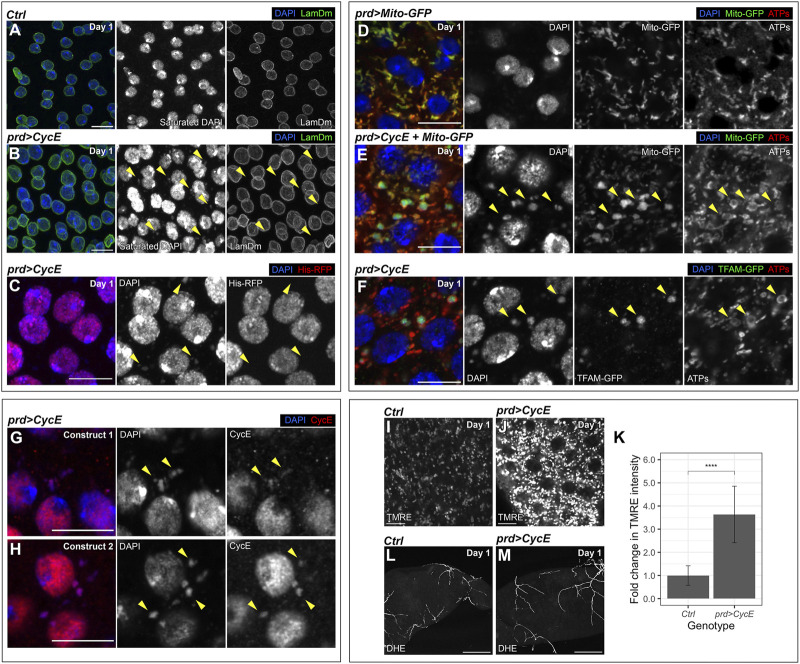
mtDNA aggregates in Cyclin E-expressing glands. **(A,B)** Confocal images of 1-day-old control (*prd-Gal4/+*) **(A)** and *prd > CycE* **(B)** glands stained with anti-Lamin Dm. The yellow arrowheads point to examples of extranuclear DNA. DAPI and LamDm are shown in blue and green, respectively. Note that the Gal80ts system was used in this experiment. Scale bars, 10 µm. **(C)** Confocal image of a 1-day-old *prd > CycE* gland containing a transgene that expresses an RFP-tagged histone. The yellow arrowheads point to examples of extranuclear DNA. DAPI and His-RFP are shown in blue and red, respectively. Note that the Gal80ts system was used in this experiment. Scale bar, 10 µm. **(D,E)** Confocal images of 1-day-old control (*prd > Mito-GFP*) **(D)** and *prd > CycE + Mito-GFP* **(E)** glands stained with anti-ATP synthase (ATPs). The yellow arrowheads point to examples of mitochondrial DNA aggregates. DAPI, Mito-GFP and ATPs are shown in blue, green, and red, respectively. Note that the Gal80ts system was used in this experiment. Scale bars, 10 µm. **(F)** Confocal image of a 1-day-old *prd > CycE* gland containing a GFP-tagged TFAM and stained with anti-GFP and anti-ATP synthase. Arrowheads point to examples of mitochondrial DNA aggregates. DAPI, TFAM-GFP and ATPs are shown in blue, green, and red, respectively. Note that the Gal80ts system was used in this experiment. Scale bar, 10 µm. **(G,H)** Confocal images of 1-day-old *prd > CycE* accessory glands, each overexpressing Cyclin E from a different transgene, and stained with anti-CycE. Construct 1 corresponds to *UAS-CycE* and construct 2 corresponds to *UAS-CycE-3xHA.* The yellow arrowheads point to examples of mitochondrial DNA aggregates containing Cyclin E. DAPI and Cyclin E are shown in blue and red, respectively. Note that the Gal80ts system was used in this experiment. Scale bars, 10 µm. **(I–K)** Confocal images of 1-day-old control (*prd-Gal4/+*) **(I)** and *prd > CycE* **(J)** accessory glands stained with TMRE, alongside their TMRE intensity quantification expressed as the relative fold change compared to the control **(K)**. In **(I,J)**, TMRE is shown in grays. Data shown in **(K)** are mean ± SD. Statistical analysis was performed using an unpaired Welch’s *t*-test. ****, *p* < .0001. Note that the Gal80ts system was used in this experiment. Scale bars, 10 µm. **(L,M)** Confocal images of 1-day-old control (*prd-Gal4/+*) **(L)** and *prd > CycE* **(M)** accessory glands stained with DHE. DHE is shown in grays. Note that the Gal80ts system was used in this experiment. Scale bars, 100 µm.

During these analyses, we observed extranuclear DAPI-positive aggregates in 1-day-old Cyclin E-expressing glands. These clusters were not present in control glands (Figures 6A, B; Supplementary Figure S7). During mitosis, missegregated chromosomes can form micronuclei, which are small chromatin bodies surrounded by a nuclear envelope. Micronuclei can be central drivers of genomic instability (Zhang et al., 2015; Krupina et al., 2021). Extranuclear DNA aggregates detected in Cyclin E-expressing glands lacked a nuclear envelope, suggesting they did not correspond to micronuclei (Figure 6B). However, previous reports have shown that, in some cases, the nuclear envelope in micronuclei can collapse (Hatch et al., 2013). We confirmed that extranuclear DNA in Cyclin E-expressing glands did not correspond to micronuclei by using a histone-RFP transgene. Nuclear DNA is organized around a core of histones and histone-RFP labels nuclear DNA. We introduced a histone-RFP transgene in Cyclin E-expressing glands and observed that, although the DNA in the nuclei was positive for histone-RFP, the DNA aggregates in the cytoplasm did not contain histone-RFP (Figure 6C). These data indicate that extranuclear DAPI-positive aggregates were not composed by nuclear DNA and hence were not micronuclei.

Mitochondria, the powerhouse of the cell, are cytoplasmic organelles that have their own DNA and hence harbor a pool of extranuclear DNA. Like the extranuclear DNA observed in Cyclin E-expressing glands, mitochondrial DNA (mtDNA) lacks histones. This opened the possibility that the DNA aggregates corresponded to mtDNA. To test this hypothesis, we labelled mitochondria by using the mitochondrial markers mito-GFP and anti-ATP synthase (Rizzuto et al., 1995; Nagaraj et al., 2012). Consistently, the extranuclear DNA in Cyclin E-expressing glands was observed in organelles positive for those mitochondrial markers (Figure 6E). Extranuclear DNA was not detected in control accessory glands (Figure 6D), suggesting that the normal mtDNA quantity was below the threshold of detection. Strikingly, mtDNA aggregates were clearly observed in Cyclin E-expressing glands (Figure 6E). This suggests that Cyclin E upregulation induces a robust increase in mtDNA quantity.

mtDNA encodes genes required for oxidative phosphorylation, which are central elements controlling cellular metabolism. Metabolic changes are emerging as central cancer drivers and, consistently, defects in mtDNA can contribute to this disease (Yu, 2011; Pavlova and Thompson, 2016). mtDNA is organized in nucleoids, which are composed by a set of DNA-binding core proteins involved in mtDNA maintenance and transcription. The transcription factor A mitochondrial (TFAM) is a central nucleoid component and plays central functions modulating mtDNA copy number. Immunomicroscopy analyses have showed that TFAM colocalizes with mtDNA in nucleoids and TFAM levels reflect the overall mtDNA levels (Ekstrand et al., 2004; Kanki et al., 2004; Gilkerson et al., 2013). We utilized a TFAM-GFP fusion protein to visualize TFAM and monitor TFAM levels in Cyclin E-expressing glands. TFAM-GFP aggregates colocalizing with mtDNA were detected in accessory gland cells expressing Cyclin E (Figure 6F). Altogether, these observations suggest that Cyclin E upregulation in the accessory gland leads to an increase in the amount of mtDNA. Although the main functions of Cyclin E occur in the nucleus, a previous study showed that Cyclin E can be recruited to the mitochondria in *Drosophila* as well as in mammalian cells (Parker et al., 2015). Consistently, we observed Cyclin E protein accumulation in cytoplasmic foci in Cyclin E-expressing glands. Those foci colocalized with extranuclear DNA (Figures 6G, H), indicating that Cyclin E can accumulate in the mitochondria. This opens the possibility that Cyclin E could have a function in the mitochondria controlling mtDNA.

Given that changes in mitochondrial mtDNA can affect mitochondrial activity, we analyzed whether Cyclin E upregulation disturbed mitochondrial function in the accessory gland. The mitochondrial electron transport chain creates an electrochemical gradient that generates the mitochondrial membrane potential (MMP). MMP is a central bioenergetic factor controlling ATP synthesis and is a key indicator of mitochondrial activity (Chen, 1988). We used the tetramethylrhodamine ethyl ester (TMRE) assay to monitor MMP. TMRE is a fluorescent dye that is sequestered by active mitochondria and it can be used to assess changes in MMP (Crowley et al., 2016). By using the TMRE assay, we detected a robust increase in MMP in Cyclin E-expressing glands, as compared to controls (Figures 6I–K). Mitochondria are the main source of reactive oxygen species (ROS) in the cell. ROS, when accumulated above normal levels, can react and damage essential cellular components such as DNA, RNA, and proteins, and consequently affect basic cellular functions. Although a direct correlation between MMP and ROS production has been demonstrated in different systems, in certain conditions negative correlations have also been reported (Suski et al., 2018). To measure ROS levels in glands upregulating Cyclin E, we used dihydroethidium (DHE), a redox-sensitive dye that shows increased fluorescence intensity when oxidized (Owusu-Ansah and Banerjee, 2009). Despite the obvious differences in TMRE intensity between glands upregulating Cyclin E and control glands (Figures 6I–K), we did not observe a change in DHE between both conditions (Figures 6L, M). These results, together, suggest that Cyclin E upregulation results in the formation of mtDNA aggregates and a concomitant increase in MMP. However, these changes in mitochondria do not cause overt changes in ROS production.

Although the different analyses presented in this study used the prd-Gal4 driver as a central tool to overexpress Cyclin E, we have reproduced the main findings obtained by expressing Cyclin E in actin-Gal4-driven Cyclin E-expressing clones (Supplementary Figure S8).

## Discussion

The experiments presented here show that Cyclin E upregulation in the accessory gland compromises tissue homeostasis and leads to tissue dysplasia. We detected numerous defects associated with Cyclin E overexpression. A superficial examination revealed that these organs display a robust increase in size. Detailed analyses showed that Cyclin E-expressing cells exhibit DNA damage and an increase in DNA content driven by endocycling. Indeed, the dysplastic glands were formed by a heterogeneous cell population exhibiting nuclei of variable sizes and aberrant shapes. Associated with this, we have shown that epithelial organization is disrupted, and epithelial integrity is compromised. Finally, we observed the presence of extranuclear DNA aggregates that accumulated in the mitochondria of Cyclin E-expressing glands.

### Cyclin E upregulation and endoreplication

We have presented evidence indicating that persistent Cyclin E expression in the binucleated cells of the accessory gland triggers endoreplication, which reproduces previous observations (Leiblich et al., 2019). These findings contrasted with previous analyses in *Drosophila* demonstrating that chronic Cyclin E expression in different organs such as salivary glands and ovarian follicle cells represses the endoreplication cycle (Calvi et al., 1998; Follette et al., 1998; Weiss et al., 1998; Shcherbata et al., 2004). Those studies showed that an oscillatory pattern of Cyclin E is crucial to sustain endoreplication. The mechanistic explanation of such a requirement is that continuous Cyclin E activity inhibits the formation of pre-replication complexes, which are required for DNA replication. Our results contrast with those reports and show that, instead of repressing, Cyclin E overexpression in male accessory glands induces endoreplicative cycles. Interestingly, we found that Cyclin E protein levels varied between cells. While some cells were positively marked by anti-Cyclin E, this signal was not detected in other cells. This observation suggests that Cyclin E activity oscillates under these circumstances, which might explain the apparent discrepancy between the experiments preformed previously in salivary glands and ovarian follicle cells, and our analyses in the accessory gland. That result would suggest that, unlike salivary glands and ovarian follicle cells, the binucleated cells of the accessory gland are able to degrade ectopically overexpressed Cyclin E, which would result in an oscillatory pattern. An obvious question remains unanswered: why Cyclin E oscillatory patterns from continuous gene overexpression can be created in the accessory gland, but not in salivary glands and ovarian follicle cells? A simple explanation could be that there are different magnitudes in Cyclin E overexpression in the different experimental conditions. We could hypothesize that strong Cyclin E overexpression might lead to a saturated situation in which cells would be unable to generate a Cyclin E oscillatory pattern. Contrary to that, moderate Cyclin E overexpression could create a context in which Cyclin E could be sensitive to cell cycle regulatory elements controlling Cyclin E degradation at the end of S-phase. This would allow for periods of low Cyclin E/CDK2 activity required for DNA licensing and subsequent DNA replication.

### Cyclin E, polyploidy, and genomic instability

The induction of endoreplication by Cyclin E in *Drosophila* male accessory glands could contribute to the gradual increase in nuclear size and DNA content observed throughout the life span of those individuals. We observed nuclei that varied greatly in size ranging from normal-looking nuclei to nuclei where the DNA is organized in structures resembling polytene chromosomes, indicative of high ploidy levels. This suggests that the extent of endoreplication and ploidy in Cyclin E-expressing glands varies between cells, which could lead to genetic imbalances. Different factors might contribute to nuclear heterogeneity. Endocycling cells do not duplicate their entire DNA content, leading to the underrepresentation of late replicating sequences (Gall et al., 1971; Hammond and Laird, 1985b; Lilly and Spradling, 1996; Lilly and Duronio, 2005). Notably, the extent of S-phase truncation has been shown to be directly affected by Cyclin E dynamics (Lilly and Spradling, 1996; Leach et al., 2000). Therefore, Cyclin E overexpression should disrupt normal Cyclin E dynamics and consequently affect the degree of S-phase truncation and resulting DNA content. We have observed robust levels of DNA damage in Cyclin E-expressing cells. This will inevitably hamper the normal DNA replication process, contributing to the generation of nuclei with different DNA content. Together, variable levels of S-phase truncation and DNA damage should affect DNA replication efficiency, leading to different levels of genome duplication, ploidy, and changes in gene copy number causing gene dose imbalance. Recent studies in flies have shown that gene dose imbalance can partially reproduce the effects observed in aneuploid tissues as well as the oncogenic potential of aneuploid cells (Clemente-Ruiz et al., 2016). Besides, changes in gene copy number affect the activity of genes contributing to tumor initiation and progression, thus having a direct influence in those processes. Those results, together with the observations presented here, insinuate a mechanism by which altered Cyclin E activity can create gene dose imbalance that could promote the formation of tissue dysplasia in the accessory gland.

### Polyploidy, a potential precancerous state

Dysplasia is a broad term that can be understood as abnormal tissue overgrowth that contains aberrant cells. It is a precancerous stage in which abnormal cells are confined within the tissue and do not invade distal organs. We have shown here that Cyclin E induces tissue overgrowth where abnormal cells can be observed. We have not observed any indication of invasion or metastatic-like behavior in these glands. However, this stage could serve as an early pre-malignant condition in which the accumulation of additional defects could promote neoplastic transformation. Notably, endoreplication and polyploidy can promote genomic instability, creating a precancerous state. Endocycling cells typically block the mitotic program, and polyploidy can act as a tumor suppressor mechanism (Zhang et al., 2018). Experiments in mammals and flies, however, have shown that when mitoses are induced in polyploid cells, these are error-prone and can result in genomic instability that enhances tumor-cell heterogeneity and promotes tumor growth (Fox and Duronio, 2013; Matsumoto et al., 2021; Wang et al., 2021). We have identified Cyclin E as a signal sufficient to promote tissue dysplasia, but it is not sufficient to drive neoplasia. Establishing whether the induction of mitosis in Cyclin E-expressing glands causes malignancy and identifying the molecular signals required to induce such transformation would merit further investigation.

### Cyclin E in the mitochondria

Mitochondria are the powerhouse of the cell and thus influence numerous cellular processes. Mitochondria harbor their own genome – a circular double-stranded DNA molecule located in the mitochondrial matrix. Mitochondrial function requires that mtDNA is duplicated and segregated properly, because defects in these processes can lead to loss of mtDNA integrity and copy number, and eventually mitochondrial dysfunction (Nicholls and Gustafsson, 2018; Chapman et al., 2020). A remarkable observation was the identification of mtDNA aggregates in Cyclin E-expressing glands. We were not able to detect mtDNA in control glands, suggesting that the amount of mtDNA in that context was below the level of detection by immunostaining. In contrast, we observed obvious DNA clusters in the mitochondria of cells overexpressing Cyclin E, indicating that those mitochondria contained a higher amount of mtDNA. Therefore, Cyclin E overexpression might induce directly or indirectly mtDNA duplication, and this would suggest a new function of this cell cycle regulator. Interestingly, we detected Cyclin E protein colocalizing with those mtDNA aggregates. This opens the possibility that Cyclin E might be actively transported to this organelle where it could control mtDNA replication and hence copy number. An alternative explanation could be based on the role of Cyclin E inducing S-phase. Crosstalk between nuclear and mtDNA is essential for cell function. Recent reports suggested that mtDNA functions such as transcription and replication are coordinated with the cell cycle (Chatre and Ricchetti, 2013; Sasaki et al., 2017). The cell cycle regulatory role of Cyclin E could thus have an impact on mtDNA amount. The formation of mtDNA clusters could have profound effects on mitochondrial function, which could, in turn, influence the formation of tissue dysplasia in Cyclin E-expressing glands. Future studies will be required to determine the consequences that mtDNA aggregates have in tissue physiology and homeostasis.

## Material and methods

### *Drosophila* strains

The following strains were provided by the Bloomington *Drosophila* Stock Center (BDSC): *prd-Gal4* (1947), *UAS-CycE* (30725), *UAS-H2Av-GFP* (93904), *His-RFP* (23651), *UAS-Mito-GFP* (8443), *TFAM-GFP* (66405), *UAS-CycE RNAi* (33654), *UAS-mCD8-GFP* (5137), and *tub-G80ts* (7018)*.* The following strain was provided by Fly-ORF: *UAS-CycE-3xHA* (F001239). Other used strains were *ap-Gal4* (Calleja et al., 1996), and the yellow white mutant strain (*yellow, white*; *yw*). Unique fly stocks were generated by combining some of these strains: *prd-Gal4*, *tub-G80ts/TM3*, *UAS-CycE*; *prd-Gal4*, *tub-G80ts/TM6B*, and *ap-Gal4*, *UAS-mCD8-GFP/CyO*; *tub-G80ts/TM6B*.

### Cross maintenance and crossing schemes

For experiments without the temperature-sensitive Gal80 system, *prd-Gal4* female virgins were crossed with males from the *yw, UAS-CycE*, or *UAS-CycE RNAi* strains. Crosses were maintained at 25°C in standard conditions and flipped every 2 or 3 days. Emerging males with the desired genotype were selected and maintained in the same environmental conditions as the corresponding crosses. Accessory glands were dissected when males reached ages ranging from 1 to 30 days (in 10-day intervals), starting to count from the day pupae hatched.

For experiments with the temperature-sensitive Gal80 system, the *prd-Gal4*, *tub-G80ts/TM3* and *UAS-CycE*; *prd-Gal4*, *tub-G80ts/TM6B* strains were used. Female virgins from these strains were accordingly crossed with the *yw, His-RFP*, *UAS-Mito-GFP*, *TFAM-GFP*, *UAS-CycE-3xHA*, or *UAS-H2Av-GFP* strains. Crosses were maintained at 18°C for 2–3 days to allow flies to lay eggs and then vials were switched to 29°C to induce Gal4 expression. Emerging males with the desired genotype were selected and maintained at 29°C. Accessory glands were dissected when males reached the ages of 1 and/or 20 days, starting to count from the day pupae hatched.

For experiments with the Flp-FRT system, *hsp70-Flp;Sb/ST* female virgins were first crossed with *act-FRT > STOP > FRT-Gal4,UAS-EGFP/CyO* males. *hsp70-Flp/+;act-FRT > STOP > FRT-Gal4,UAS-EGFP/ST* female virgins were then crossed with males from the *yw* or *UAS-CycE* strains. These crosses were maintained at 25°C in standard conditions and flipped every 2 or 3 days. To both drive flipase expression and ensure there were enough Gal4-expressing cells in the accessory glands at the time of dissection, vials with late third-instar larvae or early pupae were heat-shocked once or twice (on consecutive days) for 15 min at 37°C. More specifically, the heat-shock was performed 6 and/or 7 days after the day the cross was flipped into the vial. Emerging males with the desired genotype and with clear GFP expression were selected and maintained at 25°C. Accessory glands were dissected when males reached the ages of 1 and 20 days, starting to count from the day pupae hatched.

For experiments with wing imaginal discs, *ap-Gal4,UAS-mCD8-GFP/CyO;tub-G80ts/TM6B* female virgins were crossed with males from the *yw, UAS-CycE* or *UAS-CycE RNAi* strains. Crosses were maintained at 18°C for 2–3 days to allow flies to lay eggs and then vials were switched to 29°C to induce Gal4 expression. Wing imaginal discs from third-instar wandering larvae were dissected.

### Immunofluorescence

Primary antibodies were obtained from Developmental Studies Hybridoma Bank (DSHB), Cell Signaling Technology (CST), Santa Cruz, Rockland Sciences, or Abcam. Primary antibodies at the indicated dilutions were used as follows: mouse anti-Lamin Dm (DSHB no. ADL84.12, 1:200), mouse anti-alpha-Tubulin (DSHB no. 12G10 anti-alpha-Tubulin, 1:100), rat anti-DE-Cadherin (DSHB no. DCAD2, 1:50), rabbit anti-phospho-histone 3 (CST no. 9710S, 1:100), rabbit anti-cleaved Caspase 3 (CST no. 9661S, 1:100), rabbit anti-Cyclin E (Santa Cruz no. 33748, 1:100), rabbit anti-pH2Av (Rockland no. 234234, 1:500), mouse anti-ATP synthase (Abcam no. ab14748, 1:200), and chicken anti-GFP (Abcam no. ab13970, 1:1000). Secondary antibodies were obtained from Molecular Probes. All of them were generated in goat and used at a 1:400 dilution: anti-mouse IgG-488 (no. A-11017), anti-mouse IgG-555 (no. A-21425), anti-mouse IgG-647 (no. A-21237), anti-rabbit IgG-488 (no. A-11070), anti-rabbit IgG-555 (no. A-21428), anti-rabbit IgG-647 (no. A-21246), anti-rat IgG-647 (no. A-21247), and anti-chicken IgY-488 (no. 11039). 4’,6-diamino-2-phenylindole (DAPI) was obtained from Invitrogen (no. D1306) and used at a 1:100 dilution from a 600 nM stock solution.

Male flies were dissected in cold phosphate-buffered saline (PBS) and fixed in 3.7% formaldehyde diluted in PBS for 20 min at room temperature (RT), then washed three times for 10 min in .2% Triton X-100 diluted in PBS (PBT) and blocked for at least 20 min in 3% BSA diluted in PBT, 5 mM NaCl (BBT). Samples were incubated at RT overnight, or at 4°C for around 60 h, with primary antibody diluted in BBT. Samples were then washed three times for 10 min in BBT, incubated with fluorescent secondary antibodies and DAPI for 1.5 h at RT, and washed four times for 15 min with PBT. Accessory glands were mounted in 90% glycerol in PBS containing .4% N-propyl gallate and maintained at 4°C.

### EdU staining

This protocol was adapted from the Click-iT EdU Imaging Kit (no. C10339) from Molecular Probes. Male flies were dissected in cold PBS. Accessory glands were incubated with 300 μM 5-ethynyl-2′-deoxyuridine (EdU) for 1 h at RT. As a control, *yw* third-instar larvae were also dissected and incubated in the same working solution as accessory glands. Samples were then fixed in 3.7% formaldehyde diluted in PBS for 20 min at RT, then washed three times for 10 min in PBT and blocked for at least 20 min in BBT. Optionally, samples were incubated at RT overnight, or at 4°C for around 60 h, with primary antibody diluted in BBT. Samples were then washed three times for 10 min in BBT and incubated in the Click-IT reaction solution for 1 h at RT. Samples were washed once for 10 min in BBT, stained with fluorescent secondary antibodies and DAPI for 1.5 h at RT, and washed four times for 15 min with PBT. Accessory glands and wing imaginal discs were mounted in 90% glycerol in PBS containing .4% N-propyl gallate.

### Mitochondrial membrane potential analysis

Male flies were dissected in Schneider’s medium (Sigma, no. S9895-1L, diluted in water). Accessory glands were incubated in 100 nM tetramethylrhodamine ethyl ester (TMRE) (Sigma, no. 87917-25 MG) diluted in Schneider’s medium for 20 min at 25°C and 300 rpm. Glands were then washed for 5 min with Schneider’s medium, rinsed briefly with PBS, and mounted in Schneider’s medium before imaging. Samples were immediately imaged after mounting. Unless stated otherwise, all steps were performed at RT.

### ROS production analysis

This protocol was adapted from Owusu-Ansah et al. (2008). Male flies were dissected in Schneider’s medium. A 30 μM dihydroethidium (DHE) (Invitrogen no. D11347) working solution was prepared immediately after dissection. For this, the dye was first dissolved in anhydrous DMSO to obtain a 30 mM solution. This 30 mM DHE solution was further diluted in Schneider’s medium to obtain the 30 μM working solution. Accessory glands were incubated in this 30 μM DHE solution for 5 min. Samples were then washed three times for 5 min in Schneider’s medium, fixed for 5 min in 7% formaldehyde diluted in PBS, and rinsed once in PBS. Accessory glands were mounted in 90% glycerol in PBS containing .4% N-propyl gallate before imaging. Samples were immediately imaged after mounting. All steps were performed at RT.

### Image processing and data analysis

Images were taken using a Leica SP8 confocal microscope and analyzed with the LASX and Fiji software. In all figures (except for Figures 1L, 6D–I, Supplementary Figures S3N, O, S4E, S8O–Q), Z-stacks were imaged, and a maximum intensity projection was applied with Fiji to obtain the final image shown here. In Figures 1L, 6D–I and Supplementary Figures S3N, O, S4E, S8O–Q, only single slices were imaged. Graphs and statistical analyses were performed with R software. The exact procedures for quantification are explained in the sections below. Figures were prepared with Adobe Illustrator and Adobe Photoshop.

### Quantification of accessory gland size

Confocal Z-stack images of accessory glands were taken with the Leica SP8 confocal microscope. Images were uploaded to Fiji and a maximum intensity projection was applied. Individual accessory glands were selected with the polygon selection tool. Accessory gland area was measured with the “measure” function. At least 15 accessory glands were quantified per condition.

### Quantification of nuclear size

Confocal Z-stack images of accessory glands taken with the Leica SP8 confocal microscope. Images were uploaded to Fiji and a maximum intensity projection was applied. To quantify nuclear area, the DAPI channel was selected, and a threshold was set after applying a “Gaussian blur” of 2. The functions “fill holes” and “watershed” were used to properly segment the nuclei. The “analyze particles” option was then used to quantify individual nuclear area. At least 4 accessory glands were imaged per condition for this measurement. At least 220 nuclei were quantified per condition.

### Quantification of pH2Av intensity

Confocal Z-stack images of accessory glands stained with anti-pH2Av were taken with the Leica SP8 confocal microscope. Images were then uploaded to Fiji, a maximum intensity projection was applied and the channels were split. Then, nuclei were segmented and added to the ROI manager. For this purpose, the DAPI channel was selected, and a threshold was set after applying a “Gaussian blur” of 2. The functions “fill holes” and “watershed” were used to properly segment the nuclei. The “analyze particles” option was then used to add the nuclei to the ROI manager. Finally, the pH2Av intensity of each individual nucleus was quantified. For this purpose, the pH2Av channel was selected and the ROI selections were overlayed. The “mean gray value” was determined by using the “measure” function within the ROI manager. For each image, the background pH2Av intensity was measured and subtracted from the nuclear values of pH2Av intensity. At least 3 accessory glands were imaged per condition for this measurement. At least 165 nuclei were quantified per condition.

### Quantification of the standard deviation of Lamin Dm intensity

Confocal Z-stack images of accessory glands stained with anti-LamDm were taken with the Leica SP8 confocal microscope. Images were then uploaded to Fiji, a maximum intensity projection was applied and the channels were split. Then, the nuclei were segmented and added to the ROI manager. For this purpose, the LamDm channel was selected, and a threshold was set after applying a “Gaussian blur” of 2. The functions “fill holes” and “watershed” were used to properly segment the nuclei. The “analyze particles” option was then used to add the nuclei to the ROI manager. When not properly detected, nuclei with very low LamDm intensity were manually selected with the polygon selection tool and added to the ROI manager. Afterwards, the average LamDm intensity of each individual nucleus was quantified. For this purpose, the LamDm channel was also selected and the ROI selections were overlayed. The “mean gray value” was determined by using the “measure” function within the ROI manager. An average of 40 nuclei were quantified per gland. Finally, the standard deviation of the LamDm intensity was measured for each gland. At least 4 glands were quantified per condition to obtain an average standard deviation of LamDm intensity.

### Quantification of TMRE intensity

Confocal images of accessory glands stained with TMRE were taken with the Leica SP8 confocal microscope. Images were uploaded to Fiji, and a threshold was set to properly select mitochondria. The “mean gray value” of the thresholded area was determined with the “measure” function. Results were expressed as fold change of TMRE intensity relative to the control sample. At least 12 accessory glands were quantified per condition.

### Quantification of wing imaginal disc size

Confocal images of wing imaginal discs were taken with the Leica SP8 confocal microscope. Images were uploaded to Fiji and the GFP channel was selected. A threshold was set after applying a “Gaussian blur” of 5. The “analyze particles” option was then used to add the GFP-positive tissue to the ROI manager. GFP area was determined with the “measure” function within the ROI manager. Results were expressed as fold change of GFP area relative to the control sample*.* Around 18 wing imaginal discs were quantified per condition.

### Quantification of mtDNA aggregates

Confocal Z-stack 50 µm × 50 µm images of accessory glands were taken with the Leica SP8 confocal microscope. Images were uploaded to Fiji and the channels were split. The DAPI channel was selected and a “Gaussian blur” of 1-2 was applied. mtDNA aggregates were quantified by using the “3D Objects Counter” option. Here, the threshold was set at 40 and the minimum and maximum size filters were set at .01 and 1000, respectively. A minimum of 22 accessory glands were quantified per condition.

## Manuscript contribution

Cell proliferation is regulated by a network of elements with the aim of guaranteeing that growing or proliferating cells maintain a stable genome. Defects in this system can lead to genomic instability and thus compromise human health. Here, we examine the consequences of upregulating the cell cycle regulator Cyclin E in the *Drosophila melanogaster* male accessory gland. The accessory gland is the functional analog of the human prostate. This organ is composed of a postmitotic epithelium that is emerging as a powerful *in vivo* cancer system. We show that Cyclin E upregulation in this model is sufficient to drive the formation of tissue dysplasia. Cyclin E overexpression drives endoreplication and affects DNA integrity, which results in heterogeneous nuclear and cellular composition and variable degrees of DNA damage. We also show that Cyclin E-expressing cells in the accessory gland display mitochondrial DNA aggregates that colocalize with Cyclin E protein. Together, the findings presented here show that Cyclin E upregulation in postmitotic cells of the accessory gland organ causes cellular defects such as genomic instability and mitochondrial defects, eventually leading to tissue dysplasia. This study highlights novel mechanisms by which Cyclin E might contribute to disease initiation and progression.

## Data Availability

The original contributions presented in the study are included in the article/Supplementary Material, further inquiries can be directed to the corresponding author.
