# Supplementary material for: Cyclin E overexpression in the Drosophila accessory gland induces tissue dysplasia
Source: Front Cell Dev Biol. 2023 Jan 10;10:992253. doi: 10.3389/fcell.2022.992253 (PMC9871066; doi:10.3389/fcell.2022.992253)
Supplement: Supplementary file 1 [file Table1.docx]

**
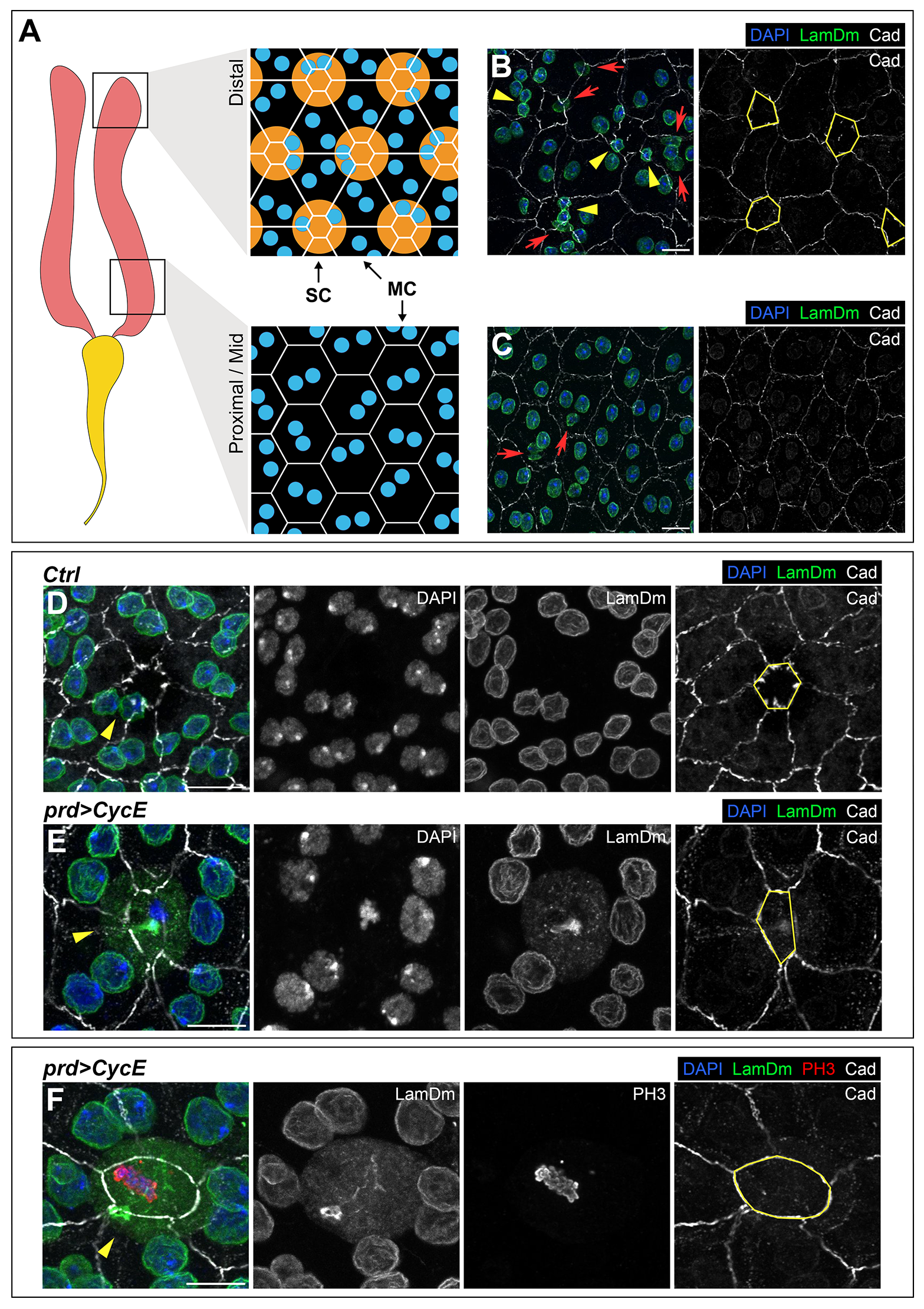
**

**Supplementary Figure S1. Stalled mitoses in secondary cells upon Cyclin E overexpression.**

**(A)** Schematic representation of main cell (MC) and secondary cell (SC) appearance and distribution in accessory glands. Main cells are drawn in black, secondary cells are drawn in orange, nuclei are drawn in blue, and DE-Cadherin is drawn in white.

**(B-C)** Confocal images that exemplify the schematic representation shown in **(A)***.* In the distal tip, there are both main and secondary cells **(B)**, whereas in the proximal or middle region of the gland, there are only main cells **(C)**. Secondary cells can be identified by their characteristic DE-Cadherin ring, outlined in yellow in the DE-Cadherin panel. The yellow arrowheads point to the nuclei of secondary cells. The red arrows point to the nuclei of muscle cells. DAPI, LamDm and Cad are shown in blue, green and grays, respectively. Scale bars, 10 µm.

**(D-E)** Confocal images of secondary cells in 1-day-old control (*prd-Gal4/+*) **(D)** and *prd>CycE* **(E)** glands stained with anti-Lamin Dm and anti-DE-Cadherin. The yellow arrowheads point to the secondary cells. The DE-Cadherin ring of these secondary cells is outlined in yellow in the DE-Cadherin panel. DAPI, LamDm and Cad are shown in blue, green and grays, respectively. Scale bars, 10 µm.

**(F)** Confocal image of a mitotic secondary cell of a *prd>CycE* gland stained with anti-LamDm, anti-PH3 and anti-DE-Cadherin. The yellow arrowhead points to the secondary cell, and the DE-Cadherin ring is outlined in yellow in the DE-Cadherin panel. DAPI, LamDm, PH3 and Cad are shown in blue, green, red and grays, respectively. Scale bar, 10 µm.


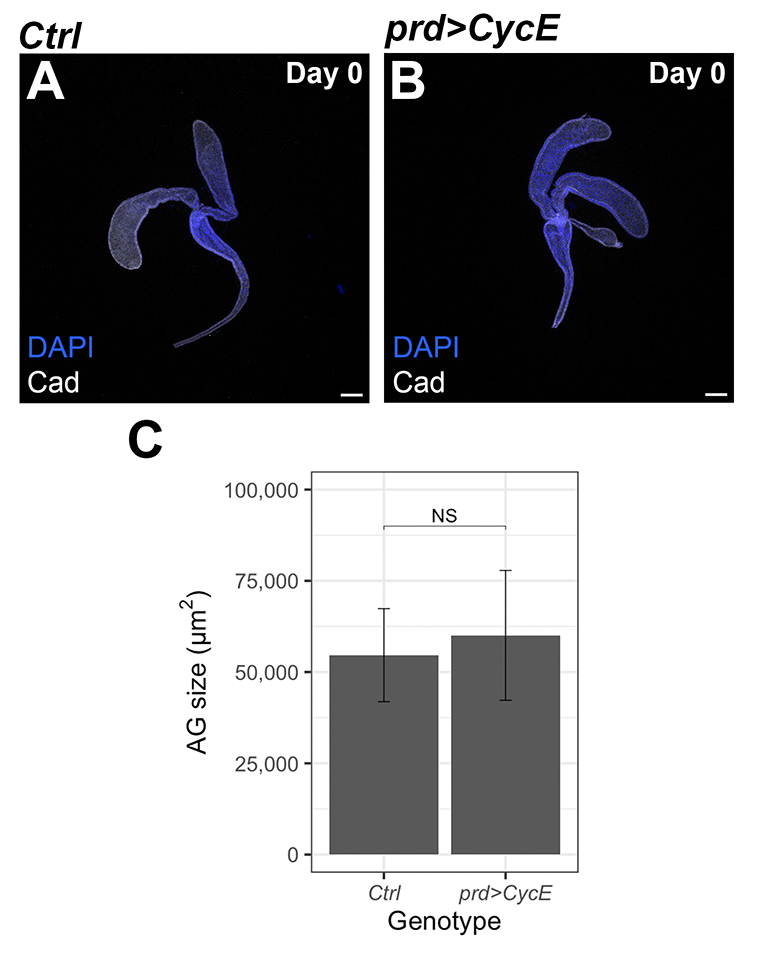


**Supplementary Figure S2. Cyclin E upregulation during accessory gland development does not affect organ size.**

**(A-C)** Confocal images of control (*prd-Gal4/+*) **(A)** and *prd>CycE* **(B)** glands just after eclosion, and their size quantification **(C)**. There were no significant differences in size between control and Cyclin E-expressing glands of males that had just eclosed. In **(A,B)***,* DAPI and Cad are shown in blue and grays, respectively. Data shown in **(C)** are mean ± SD. Statistical analysis was performed using an unpaired Student’s t-test. NS, non-significant. Scale bars, 100 μm.

**
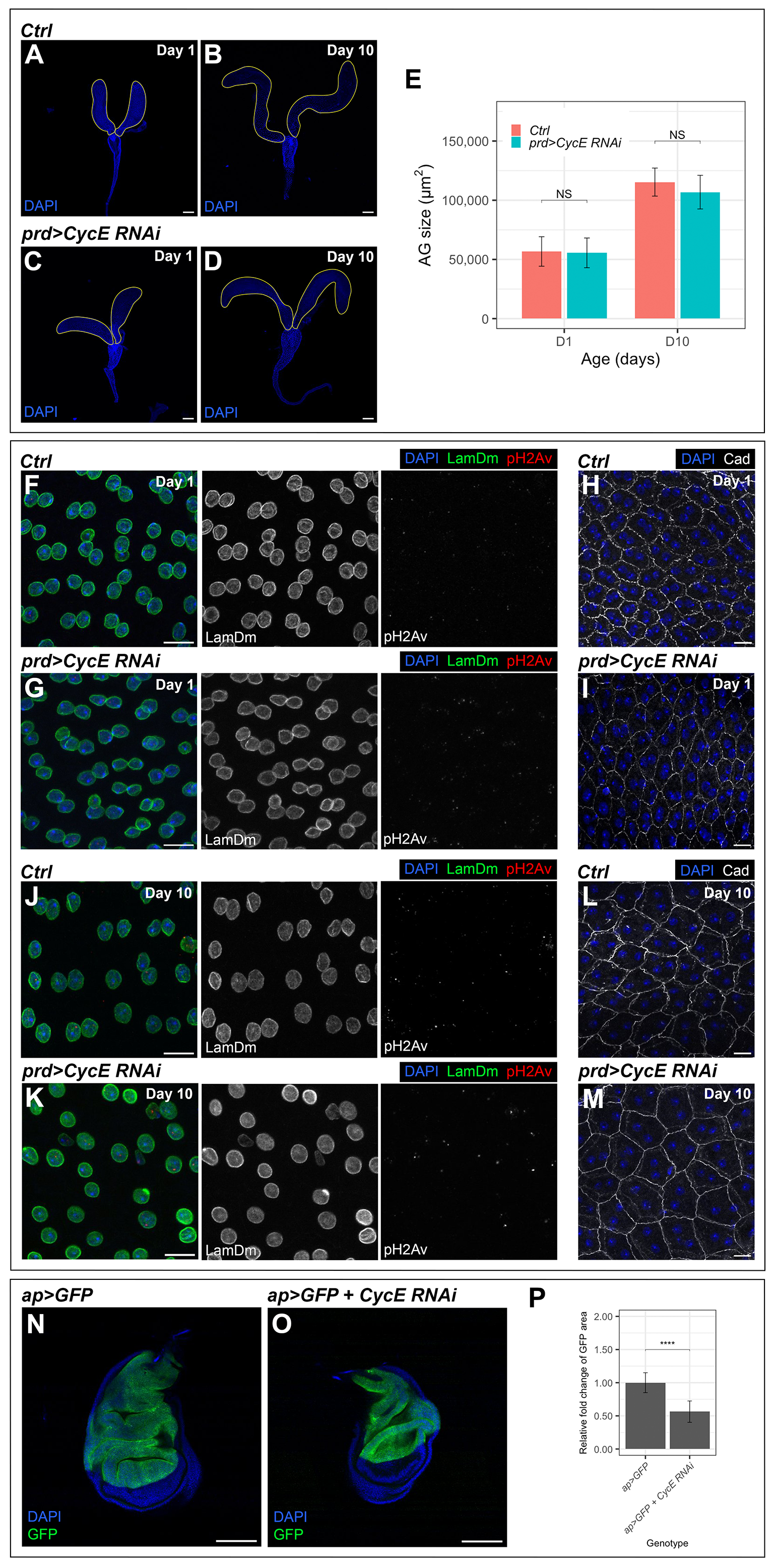
**

**Supplementary Figure S3. CycE downregulation does not cause major phenotypes in the accessory gland.**

**(A-E)** Confocal images of 1-day-old and 10-day-old control (*prd-Gal4/+*) [**(A,B)**, respectively] and *prd>CycE RNAi* [**(C,D)**, respectively] accessory glands, and their size quantification **(E)**. There were no significant differences in tissue size between control glands and glands where Cyclin E has been downregulated. In **(A-D)***,* accessory glands are outlined in yellow, and DAPI is shown in blue. Data shown in **(E)** are mean ± SD. Statistical analysis was performed using a two-way ANOVA followed by a Tukey’s HSD test. Only the relevant significant changes are shown. NS, non-significant. Scale bars, 100 µm.

**(F-G, J-K)** Confocal images of 1-day-old and 10-day-old control (*prd-Gal4/+*) [**(F,J)**, respectively] and *prd>CycE RNAi* [**(G,K)**, respectively] glands stained with anti-Lamin Dm and anti-pH2Av. No obvious changes were observed after Cyclin E downregulation in terms of nuclear and cellular appearance in neither 1-day-old nor 10-day-old glands. DAPI, LamDm and pH2Av are shown in blue, green, and red, respectively. Scale bars, 10 µm.

**(H-I, L-M)** Confocal images of 1-day-old and 10-day-old control (*prd-Gal4/+*) [**(H,L)**, respectively] and *prd>CycE RNAi* [**(I,M)**, respectively] glands stained with anti-DE-Cadherin. No obvious changes were observed after Cyclin E downregulation in terms epithelial integrity in neither 1-day-old nor 10-day-old glands. DAPI and Cad are shown in blue and grays, respectively. Scale bars, 10 µm.

**(N-P)** Confocal images of control (*ap>GFP*) **(N)** and *ap>GFP+CycE RNAi* **(O)** wing imaginal discs, and their quantification of GFP area expressed as the relative fold change compared to the control **(P)**. GFP labels the dorsal compartment, where the *ap-Gal4* driver is expressed. Albeit not having any obvious effect in accessory glands, the *CycE* RNAi impaired wing imaginal disc growth. This experiment served as a positive control for the *CycE* RNAi. In **(N,O)***,* DAPI and GFP are shown in blue and green, respectively. Data shown in **(P)** are mean ± SD. Statistical analysis was performed using an unpaired Student’s t-test. ****, p<0.0001. Note that the Gal80ts system was used in this experiment. Scale bars, 100 µm.


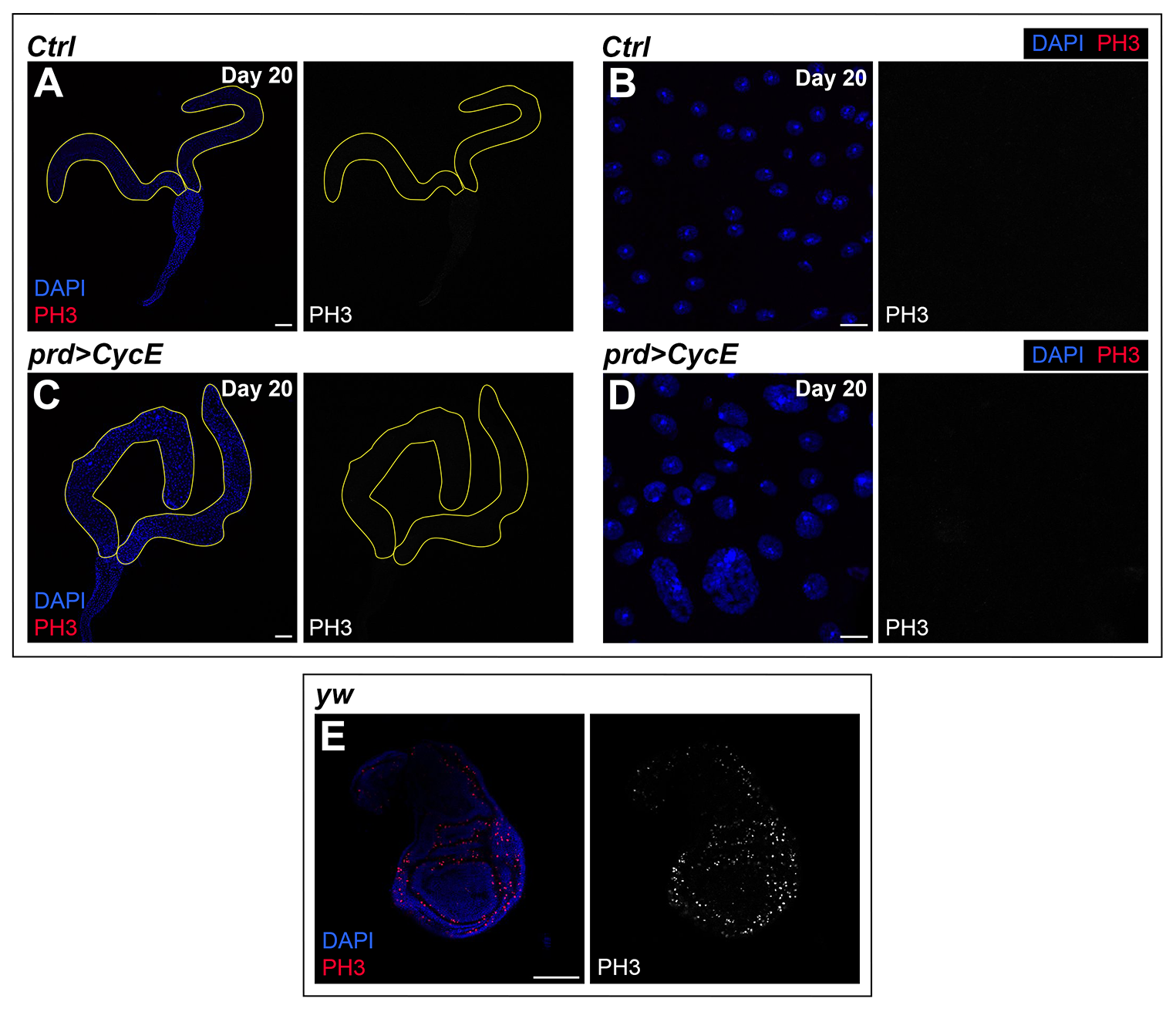


**Supplementary Figure S4. Mitoses are not detected in 20-day-old male accessory glands.**

**(A-D)** Confocal images of 20-day-old control (*prd-Gal4/+*) **(A,B)** and *prd>CycE* **(C,D)** accessory glands stained with anti-PH3. No mitoses were detected in neither control nor Cyclin E-expressing glands, as determined by the anti-PH3 staining and the nuclear shapes. In **(A,C)***,* accessory glands are outlined in yellow. DAPI and PH3 are shown in blue and red, respectively. Scale bars, 100 µm **(A,C)** and 10 µm **(B,D)**.

**(E)** Confocal image of a *yw* wing imaginal disc stained with anti-PH3. This staining served as a positive control for mitosis. DAPI and PH3 are shown in blue and red, respectively. Scale bar, 100 µm.


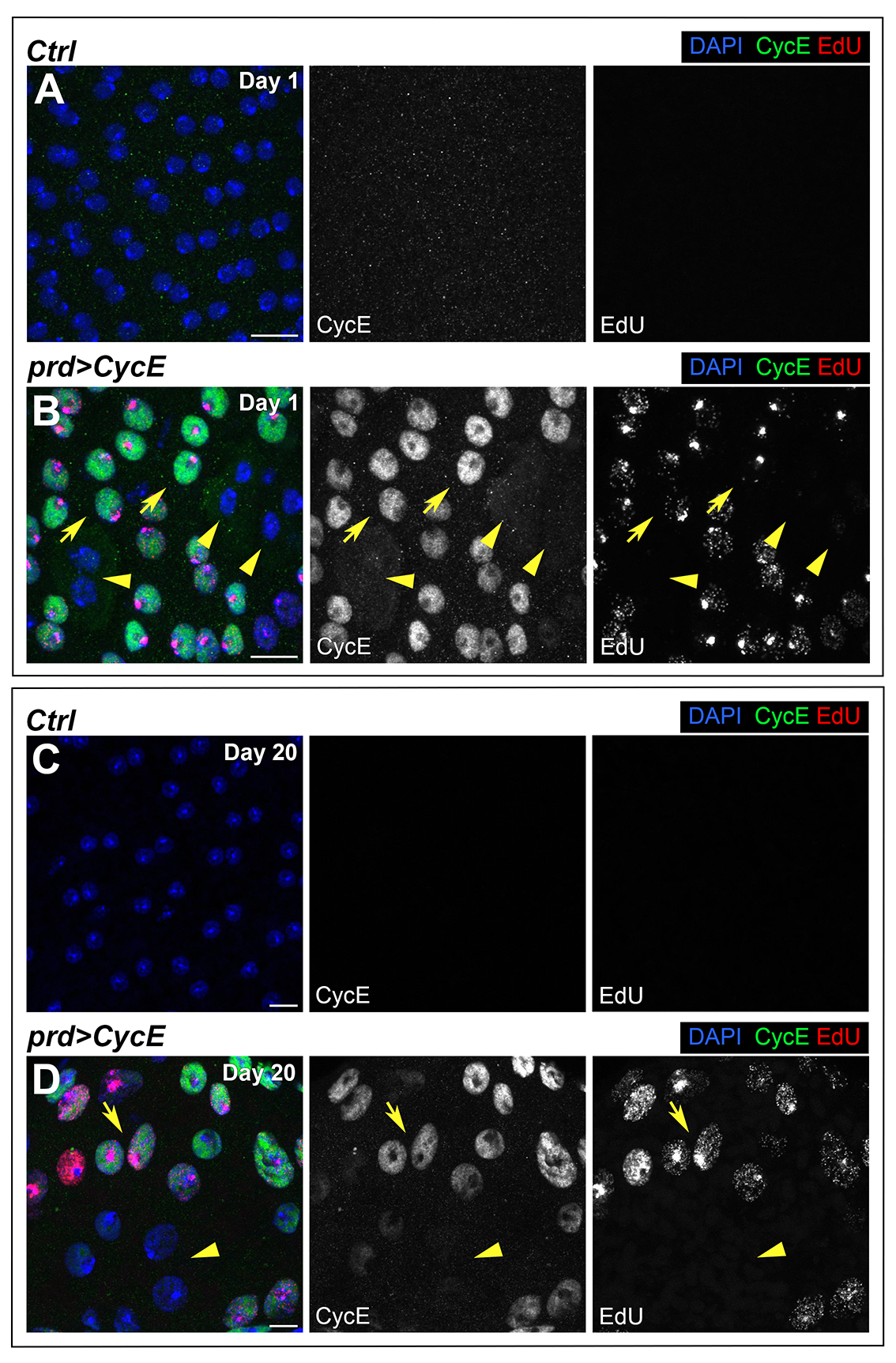


**Supplementary Figure S5. CycE and EdU incorporation.**

**(A-D)** Confocal images of 1-day-old and 20-day-old control (*prd-Gal4/+*) [**(A,C)**, respectively] and *prd>CycE* [**(B,D)**, respectively] accessory glands labeled with EdU and anti-Cyclin E. Cyclin E was not present in control glands, and they did not incorporate EdU, neither at day 1 nor at day 20. By contrast, cells in Cyclin E-expressing glands generally had high levels of nuclear Cyclin E, corresponding to a certain degree of DNA synthesis in those nuclei (yellow arrows). Cells in Cyclin E-expressing glands that did not display any Cyclin E in their nuclei did not incorporate EdU (yellow arrowheads). DAPI, CycE and EdU are shown in blue, green, and red, respectively. Scale bars, 10 µm.


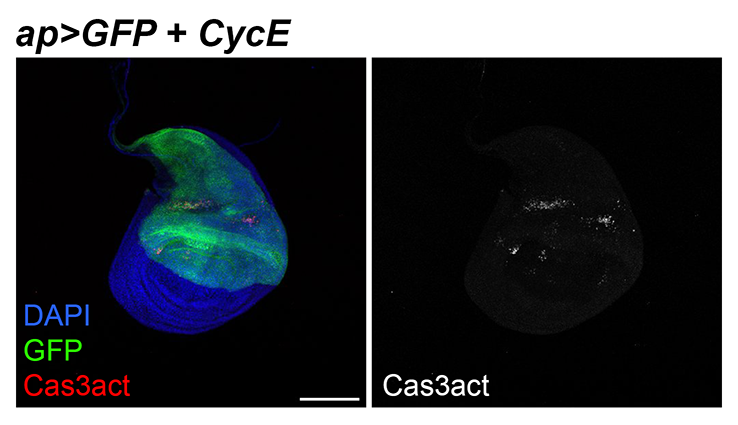


**Supplementary Figure S6. Positive control for apoptosis labeling.**

Confocal image of an *ap>GFP+CycE* wing imaginal disc stained with anti-cleaved Caspase 3. GFP labels the dorsal compartment, where the *ap-Gal4* driver is expressed. This staining served as a positive control for apoptosis. DAPI, GFP and Cas3act are shown in blue, green, and red, respectively. Note that the Gal80ts system was used in this experiment. Scale bar, 100 µm.


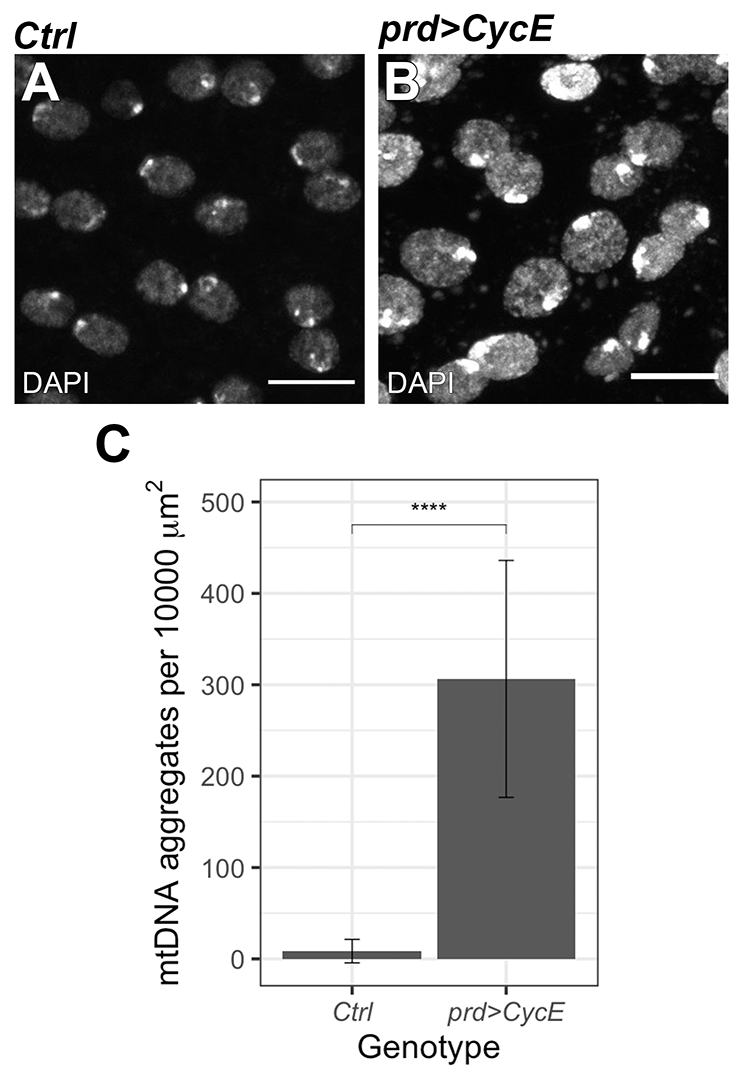


**Supplementary Figure S7. Cyclin E overexpression leads to the formation of mtDNA aggregates.**

**(A-B)** Confocal images of 1-day-old control (*prd-Gal4/+*) **(A)** and *prd>CycE* **(B)** accessory glands. DAPI is shown in grays. Note that the Gal80ts system was used in this experiment. Scale bars, 10 µm.

**(C)** Quantification of mtDNA aggregates in control (*prd-Gal4/+*) and *prd>CycE* glands as shown in **(A,B)***,* respectively. Data shown here are mean ± SD. Statistical analysis was performed using a Mann-Whitney test for unpaired data. ****, p<0.0001.


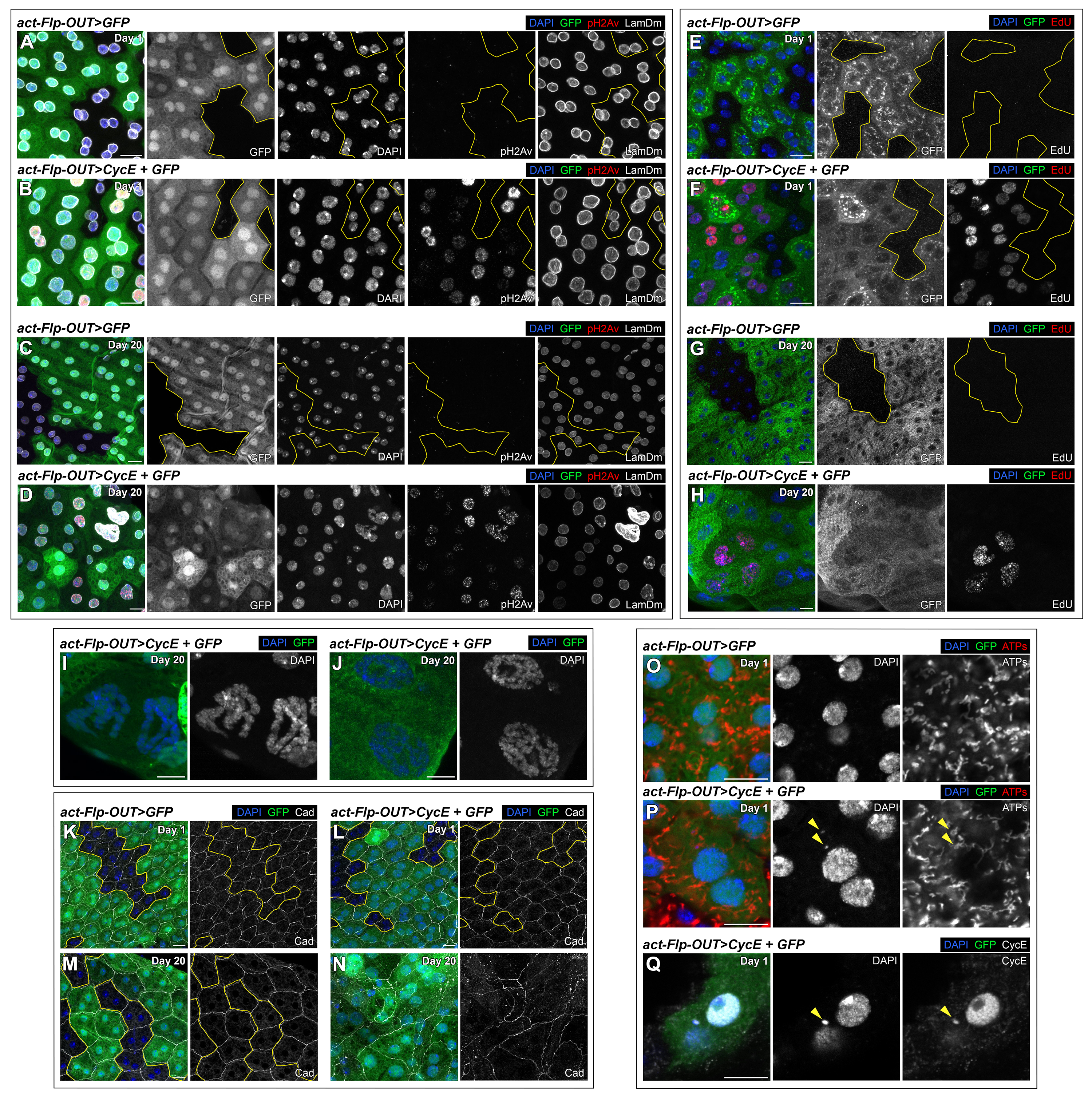


**Supplementary Figure S8. Phenotypes associated to Cyclin E overexpression in Flp-OUT clones.**

**(A-D)** Confocal images of 1-day-old and 20-day-old control (*act-Flp-OUT>GFP*) [**(A,C)**, respectively] and *act-Flp-OUT>CycE+GFP* [**(B,D)**, respectively] glands stained with anti-pH2Av and anti-Lamin Dm. Gal4-expressing cells can be identified by GFP, and the borders between GFP-positive and GFP-negative cells are outlined in yellow. Cyclin E overexpression under *act-Flp-OUT-Gal4* control led to an increase in nuclear size and DNA damage already at day 1. These phenotypes persisted and even became more apparent at day 20, when nuclei displayed more heterogeneous sizes, more heterogeneous Lamin Dm levels, and varying amounts of DNA damage. Nevertheless, these phenotypes in aging glands were less dramatic compared to the ones obtained with *prd-Gal4.* DAPI, GFP, pH2Av and LamDm are shown in blue, green, red, and grays, respectively. Scale bars, 10 µm.

**(E-H)** Confocal images of 1-day-old and 20-day-old control (*act-Flp-OUT>GFP*) [**(E,G)**, respectively] and *act-Flp-OUT>CycE+GFP* [**(F,H)**, respectively] glands labeled with anti-GFP and EdU. Gal4-expressing cells can be identified by GFP, and the borders between GFP-positive and GFP-negative cells are outlined in yellow. Cyclin E overexpression in these glands caused cells to actively replicate their DNA, especially at day 1. At day 20, EdU incorporation in Cyclin E-expressing glands was restricted to very few cells, which could be due to the *act-Flp-OUT-Gal4* driver being weaker than the *prd-Gal4* driver. DAPI, GFP and EdU are shown in blue, green, and red, respectively. Scale bars, 10 µm.

**(I-J)** Confocal images of Gal4-expresing cells in 20-day-old *act-Flp-OUT>CycE+GFP* glands showcasing polytene chromosomes. The presence of polytene chromosomes further confirmed cells were undergoing endoreplication. DAPI and GFP are shown in blue and green, respectively. Scale bars, 10 µm.

(**K-N)** Confocal images of 1-day-old and 20-day-old control (*act-Flp-OUT>GFP*) [**(K,M)**, respectively] and *act-Flp-OUT>CycE+GFP* [**(L,N)**, respectively] glands labeled with anti-DE-Cadherin. Gal4-expressing cells can be identified by GFP, and the borders between GFP-positive and GFP-negative cells are outlined in yellow. Epithelial integrity in Cyclin E-expressing cells was not affected in 1-day-old glands. In 20-day-old glands, however, Cyclin E-overexpressing cells had an aberrant DE-Cadherin expression pattern and had lost their epithelial integrity. DAPI, GFP and Cad are shown in blue, green, and grays, respectively. Scale bars, 10 µm.

**(O-P)** Confocal images of Gal4-expresing cells in control (*act-Flp-OUT>GFP*) **(O)** and *act-Flp-OUT>CycE+GFP* **(P)** glands at day 1 stained with anti-ATP synthase. In contrast to control cells, Cyclin E-expressing cells displayed mtDNA aggregates (yellow arrowheads). Interestingly, the presence of mtDNA aggregates in cells that expressed Cyclin E under *act-Flp-OUT-Gal4* control was ostensibly less frequent than in those expressing Cyclin E under *prd-Gal4* control. This could also be explained by a lower strength of the *act-Flp-OUT-Gal4* driver compared to the *prd-Gal4* driver. DAPI, GFP and ATPs are shown in blue, green, and red, respectively. Scale bars, 10 µm.

**(Q)** Confocal image of a Gal4-expressing cell in an *act-Flp-OUT>CycE+GFP* accessory gland at day 1 stained with anti-Cyclin E. As occurred with the *prd-Gal4* driver, mtDNA aggregates (yellow arrowhead) contained Cyclin E. DAPI, GFP and CycE are shown in blue, green, and grays, respectively. Scale bar, 10 µm.
